# Comparative pathogenicity of CA1737/04 and Mass infectious bronchitis virus genotypes in laying chickens

**DOI:** 10.3389/fvets.2024.1338563

**Published:** 2024-02-28

**Authors:** Ahmed Ali, Muhammad Farooq, Danah Altakrouni, Shahnas M. Najimudeen, Mohamed S. H. Hassan, Ishara M. Isham, Adel A. Shalaby, Rodrigo A. Gallardo, Mohamed Faizal Abdul-Careem

**Affiliations:** ^1^Faculty of Veterinary Medicine, University of Calgary, Calgary, AB, Canada; ^2^Department of Pathology, Faculty of Veterinary Medicine, Beni-Suef University, Beni Suef, Egypt; ^3^Department of Avian and Rabbit Medicine, Faculty of Veterinary Medicine, Assiut University, Assiut, Egypt; ^4^Department of Population Health and Reproduction, School of Veterinary Medicine, University of California, Davis, Davis, CA, United States

**Keywords:** infectious bronchitis virus, Canada, hen, pathogenicity, tissue tropism

## Abstract

Infectious bronchitis virus (IBV) is a respiratory virus causing atropism in multiple body systems of chickens. Recently, the California 1737/04 (CA1737/04) IBV strain was identified as one of the circulating IBV variants among poultry operations in North America. Here, the pathogenicity and tissue tropism of CA1737/04 IBV strain in specific-pathogen-free (SPF) hens were characterized in comparison to Massachusetts (Mass) IBV. In 30 weeks-old SPF hens, Mass or CA1737/04 IBV infections were carried out, while the third group was maintained as a control group. Following infection, we evaluated clinical signs, egg production, viral shedding, serology, necropsy examination, and histopathology during a period of 19 days. Also, certain tissue affinity parameters were investigated, which involved the localization of viral antigens and the detection of viral RNA copies in designated tissues. Our findings indicate that infection with CA1737/04 or Mass IBV strain could induce significant clinical signs, reduced egg production, and anti-IBV antibodies locally in oviduct wash and systemically in serum. Both IBV strains showed detectable levels of viral RNA copies and induced pathology in respiratory, renal, enteric, and reproductive tissues. However, the CA1737/04 IBV strain had higher pathogenicity, higher tissue tropism, and higher replication in the kidney, large intestine, and different segments of the oviduct compared to the Mass IBV strain. Both IBV strains shed viral genome from the cloacal route, however, the Mass IBV infected hens shed higher IBV genome loads via the oropharyngeal route compared to CA1737/04 IBV-infected hens. Overall, the current findings could contribute to a better understanding of CA1737/04 IBV pathogenicity in laying hens.

## 1 Introduction

Infectious bronchitis (IB) is a highly contagious respiratory disease and is caused by the infectious bronchitis virus (IBV), which primarily infects chickens (1). IBV is a gammacoronavirus belonging to the family of *Coronaviridae*. IBV is a primary respiratory pathogen, it initially replicates in the epithelial cells of the upper respiratory tract of chickens causing respiratory manifestations such as gasping, coughing, sneezing, tracheal rales, and nasal discharge (2). Furthermore, it has been known that IBV could target lung macrophages (3) and monocytes (4), a potential reason why it disseminates beyond the respiratory tract. In addition to respiratory disease, IBV infection may lead to renal, reproductive, or gastrointestinal pathologies depending on the infecting IBV strain or genotype (1). Various serotypes and variants of IBV are circulating all over the world, this is attributed to genetic diversity as a result of mutation and recombination events (5). Despite extensive vaccination against IBV, there is a poor cross-protection between heterologous strains (6).

A majority of IBV nephropathogenicity cases have been described in broilers. Several QX-type IBV strains have been associated with renal disease in Asia, Europe, and Africa (7–9). In the United States of America (USA), IBV Gray, PA/Wolgemuth/98, PA/171/99 and 98, and Delmarva (DMV/1639/11) are known to induce renal pathology (10–12). Nevertheless, some IBV strains have been reported to also induce nephropathogenicity in laying hens (13, 14). The nephropathogenic strains of IBV induce nephritis, which looks grossly like swollen and pale kidneys with distended tubules and ureters as a result of urate accumulation (15). In the first documented report of nephropathogenic IBV, coexisting clinical signs of enteritis were described in broilers (10). Since that time, it has been documented that IBV can infect various epithelial cells along the digestive tract prolonging viral shedding (16–18).

The pathogenicity of IBV in reproductive tissues in chickens is primarily determined by the age of birds at the time of infection, their immune status, and the IBV strain involved (19). False layer syndrome due to cystic oviduct can be produced by some IBV strains when infections occur before 2 weeks of age (20). Infection of adult layers may be associated with a range of clinical signs varying from eggshell defects to egg production declines (21). IBV strains such as M41, Aust T, and QX-like have been identified to cause reproductive pathology with subsequent egg production and quality deteriorations (22–24).

There have been numerous strains of IBV isolated in the USA since the 1930s, most belonging to the GI-9 lineage (like ArkDPI), GI-17 lineage (like DMV/1639), and GI-25 lineage [like California 1737/04 (1737/04)] (25–27). At present, GI-17 (like DMV/1639) is the most common variant of IBV circulating among poultry in the US; it was first isolated in 2011 and started causing significant diseases in 2014/2015 (26). In Canada, IBV strains could be categorized into four main groups including unique Canadian variants (strain Qu-mv), classic [vaccine-like viruses such as Connecticut (Conn) and Massachusetts (Mass)], variant strains of US origin (CA1737, California 99, CU_82792, Pennsylvania 1220/98, Pennsylvania Wolf/98, and DMV/1639), and non-Canadian/non-US variants or European strains (793/B strain) (28, 29).

Currently, there are two main IBV genotypes affecting layer operations in Canada. The Mass-type variants mostly impact commercial layers in Western Canada (3). The Eastern Canadian layer flocks are predominantly affected by DMV/1639-type variants (29). Since 2012, CA1737/04 strain has been detected in Ontario poultry flocks (28). In 2004, CA1737/04 IBV was first isolated from broilers (46 days-old) and pullets (4 to 6 weeks-old) in California with a history of respiratory distress and nephritis, respectively (30). Interestingly, CA1737/04 was detected in laying hens presenting with cystic oviducts on a commercial farm located in Arizona (31). To date, no controlled experimental studies have been conducted in laying hens to evaluate the pathogenicity of CA1737/04. Consequently, a comprehensive experiment was carried out in specific-pathogen-free (SPF) White Leghorn layers to examine the pathogenicity and tissue tropism of CA1737/04. This experimental infection was compared against infection with a well-known Mass strain linked to egg production issues in Western Canada (3).

## 2 Materials and methods

### 2.1 Viral strains

The Mass IBV isolate (designated as 15AB-01) of the GI-1 lineage was isolated from layers in Alberta with a history of altered egg production (3), while the CA1737/04 IBV was isolated from tracheal swabs obtained from broiler chickens (25 days-old) with respiratory manifestations in California, USA. The CA1737/04 IBV was received from the Department of Population Health and Reproduction, School of Veterinary Medicine, University of California (Davis, CA, USA). Genotype identification was performed by comparing amplified fragments of the spike 1 gene with sequences available in the NCBI GenBank database. The current IBV isolate displayed more than 97% similarity with CA1737/04 (EU925393) (32) which belongs to the GI-25 (33). Propagation of both viral stocks was achieved by inoculating SPF embryonated chicken eggs via the allantoic cavity at 9 to 11 days of age. The 50% embryo infectious dose (EID_50_) of the fourth and third passages of Mass and CA1737/04 was determined based on Reed and Muench’s method (34), respectively.

### 2.2 Laying chickens and ethics statement

Twenty-four-weeks-old SPF laying hens (White Leghorn) were obtained from the Canadian Food Inspection Agency (CFIA), Ottawa, Ontario, and transferred to negative pressure rooms at the University of Calgary’s Veterinary Science Research Station (VSRS). Chickens were allowed 6 weeks to acclimate and stabilize the egg production. Throughout the experiment, the chickens received feed and water *ad libitum*. The light schedule comprised 8 h of darkness/16 h of light. All chickens utilized in this study were cared for and the experimental procedures were performed following approval of the Veterinary Science Animal Care Committee (VSACC) at the University of Calgary (protocol number AC19-0011).

### 2.3 Experimental groups and viral infection

A total of fifty SPF-laying chickens were randomly divided into three groups, a separate room for each group. At 30 weeks old, two groups were infected with 1 × 10^6^ EID_50_ of IBV strains Mass and CA1737/04. A total volume of 150 μL of allantoic fluid containing either the Mass or CA1737/04 IBV strain was used with 80 μL intratracheally, 35 μL intranasally, and 35 μL via the ocular route under isoflurane anesthesia. Therefore, there were two IBV-infected groups: Mass-infected (*n* = 16) and CA1737/04-infected (*n* = 18). While the third group was mock-infected with 150 μL sterile phosphate-buffered saline (PBS; Life Technologies Corporation, Grand Island, NY, United States), this group was maintained as the uninfected control (*n* = 16). Following IBV infection, all birds were monitored daily for clinical signs and egg production until 19 and 18 days post-infection (dpi), respectively.

### 2.4 Evaluation of clinical signs and egg production performance

In all groups, clinical signs were observed twice daily after the experimental infection of IBV and categorized into four severity scores (ranging from 0 to 3) as assessed previously (35). Briefly, general clinical signs including ruffled feathers, huddling together to a heat source, depression with a lowered head, and droopy wings received a score of 1. Respiratory signs were scaled as follows: score (0) (absence of signs), score (1) mild (increased respiration but beaks remained closed), score (2) moderate (increased respiration with open beaks, coughing, sneezing, watery eyes, and nasal discharge), while score (3) was described to severe signs (marked gasping). For each group, the daily average clinical sign scores were reported and calculated using the aforementioned scoring system.

From 3 days pre-infection and 18 dpi, the egg production was recorded individually for each group. Egg laying performance was evaluated as the percentage of egg production/group/three-day interval (100% = 1 egg/hen/day).

### 2.5 Sampling and necropsy examination

At 3, 7, 9, 13, and 19 dpi, oropharyngeal (OP) and cloacal (CL) swabs were collected and preserved in PBS containing 2% of fetal calf serum and 2% of penicillin and streptomycin (Gibco, Carlsbad, CA, United States), aliquoted and kept at −80°C until use. Blood was collected from the wing veins of each bird at 9, 13, and 19 dpi to monitor serum anti-IBV titers. At 9 dpi, six birds from each group were randomly selected and euthanized following over-inhalation of isoflurane anesthesia and cervical dislocation to inspect gross lesions and collect samples. Similarly, the rest of the birds in all experimental groups were euthanized at 19 dpi. Tissue portions representing various body systems such as trachea, lung, kidney, cecal tonsils, duodenum, colorectum, ovary, and oviduct (magnum, isthmus, uterus) were collected in in RNA Save^®^ (Biological Industries, Beit Haemek, Israel) for IBV genome load quantification. Specimens from the above-mentioned tissues with or without cecal tonsils were preserved in 10% neutral buffered formalin (VWR International, Edmonton, AB, Canada) for histopathological or immunohistochemical examination, respectively.

Gross lesions were examined at 9 and 19 dpi with emphasis on the reproductive tract, i.e., ovaries and oviducts, as well as the length of the oviduct in all birds was measured. In order to collect oviduct washes, 10 mL of cold PBS was infused and gently massaged throughout the entire length of the oviduct (36), then aliquoted and stored at −80°C until processing.

### 2.6 Techniques

#### 2.6.1 Monitoring of anti-IBV antibodies

Blood samples at 9, 13, and 19 dpi and oviductal washes at 9 and 19 dpi were collected to measure the antibody responses against IBV using a commercial ELISA kit (IDEXX Laboratories, Inc., Westbrook, ME, United States). The assay was performed based on the manufacturer’s protocol. Titers greater than 396 (cut-off) were considered positive.

#### 2.6.2 IBV genome load quantification

Total ribonucleic acid (RNA) was extracted from the tissues and swabs using Trizol^®^ reagent (Invitrogen Canada Inc., Burlington, ON, Canada) following the manufacturer’s recommendations. RNA concentration was measured with a Nanodrop 1,000 spectrophotometer (ThermoScientific, Wilmington, DE, United States). To synthesize the cDNA from swabs and tissues, we used 1,000 and 2,000 ng of RNA, respectively, using random primers (High-Capacity Reverse Transcription Kit^™^, Applied Biosystems, Invitrogen Canada Inc., Burlington, ON, Canada). The IBV genome load quantification was performed by real-time quantitative polymerase chain reaction (RT-qPCR) test using the CFX 96-c1000 Thermocycler (Bio-Rad Laboratories, Mississauga, ON, Canada). The RT-qPCR test was performed based on a SYBR^®^ Green Master Mix (Invitrogen, Burlington, ON, Canada). Each reaction was adjusted to 20 μL net volume, including 10 μL of SYBR Green master mix, 100 ng of cDNA per sample, and forward and reverse specific primers (0.5 μL for each). The primers used in this RT-qPCR assay targeted the nucleocapsid (N) gene of IBV as described previously (37). In order to quantify the absolute number of IBV genome copies, a standard curve was used using six ten-fold serial dilutions (10^7^–10^2^) of in-house prepared plasmids bearing the IBV-N gene (37).

#### 2.6.3 Histopathology

Following each necropsy examination (9 and 19 dpi), the formalin-fixed tissue specimens from the trachea, lung, kidney, duodenum, colorectum, ovary, and oviduct (magnum, isthmus, and uterus) were processed with standard histological techniques, embedded in paraffin, and cut into 4 μm sections mounted on glass slides. The cut sections were then stained with haematoxylin and eosin (H&E) at the Diagnostic Services Unit (DSU) of the University of Calgary according to a previously described procedure (38). The stained tissue sections were visualized under light microscopy (Olympus BX51, Center Valley, PA, United States) for lesions linked to IBV infection. The histopathological lesions were listed (Table 1) and scored according to Benyeda et al. (39) with some modifications. The assigned lesions (Table 1) were categorized as follows: no change (0), mild (1), moderate (2), or severe (3), as published previously (40, 41).

**Table 1:** Histopathological lesions detected and scored.

Tissue	Microscopic lesions
Trachea	Epithelium Epithelial hyperplasia Epithelial thinning Epithelial loss Deciliation Lamina propria Inflammatory cell infiltrations Dilated mucosal glands Congestion and/or hemorrhage
Lung	Peri-bronchitis Inflammatory cell infiltrations in the interstitial tissue Circulatory disturbances (hyperemia, edema, and hemorrhage in the interstitial tissue)
Kidney	Necrosis of ducto-tubular epithelium Inflammatory cell infiltrations in the interstitial tissue Renal tubular dilatation Urate like or mineral deposits
Gastrointestinal tract (duodenum and colorectum)	Necrosis of villus epithelium Inflammatory cell infiltrations in subepithelial tissue
Ovary	Necrosis of ovarian covering epithelium Heterophilic and/or mononuclear cell infiltrations in the cortical stroma Congestion and edema in the cortical stroma
Oviduct (magnum, isthmus, and uterus)	Epithelial cell necrosis Loss of cilia Inflammatory cell infiltrations in lamina propria Edema and congestion in lamina propria

#### 2.6.4 Immunohistochemistry

Immunohistochemistry (IHC) was performed on paraffin sections prepared from the tissues (trachea, lung, kidney, cecal tonsils, duodenum, colorectum, ovary, magnum, isthmus, and uterus) collected at 9 and 19 dpi. Following deparaffinization, the tissue sections were treated with 3% hydrogen peroxide in methanol to block the endogenous peroxidase activity. The epitopes were retrieved by microwaving the tissue sections in 10 mM citrate buffer (PH 6.0) at 850 Watts for 10–15 min. After treating the slides, they were cooled at room temperature for 30 minutes (min). Non-specific background staining was quenched by incubating the sections in the blocking buffer composed of 2.5% goat serum in PBS for 1 h at room temperature. Detection of IBV was achieved using 1: 400 mouse anti-IBV monoclonal antibody specific for the N-protein (Novus Biological, Bio-Techne, Toronto, ON, Canada) in the blocking buffer with an overnight incubation at 4°C. As a secondary antibody, biotinylated goat anti-mouse IgG (H + L) (Vector Laboratories, Burlingame, CA, United States) was employed. The antibody binding was detected using an avidin-biotin-peroxidase complex (ABC) system (Vectastain^®^ ABC kit, Vector Laboratories, Burlingame, CA, United States) following the manufacturer’s instructions. Visualization of antigen localization was performed by incubating the sections in 3,3-diaminobenzidine-H_2_O_2_ solution (DAB substrate kit for peroxidase, Vector Laboratories, Burlingame, CA, United States). Eventually, the tissue sections were counter-stained with Gill’s hematoxylin (Electron Microscopy Sciences, Hatfield, PA, United States) and mounted with a toluene-based liquid mounting medium. Five microscopic fields with high IBV positive cells per tissue section were captured at 20× magnification. The percentage of IBV positive cells per field was calculated using an online dataset known as DeepLIIF (42).

#### 2.6.5 Data analysis

The proportions of a three-day interval of egg production were compared between the groups using Fisher’s exact test. The group differences in all other tested parameters were analysed by the Kruskal–Wallis test followed by Dunn’s multiple comparison test at each time point. In order to assess the normality of the data, the D’Agostino–Pearson test was employed. GraphPad Prism 9.4.1 software (GraphPad Prism Software, San Diego, California, United States) was used to perform all statistical tests and graphs.

## 3 Results

### 3.1 Clinical manifestations and egg production

**Figure 1:**
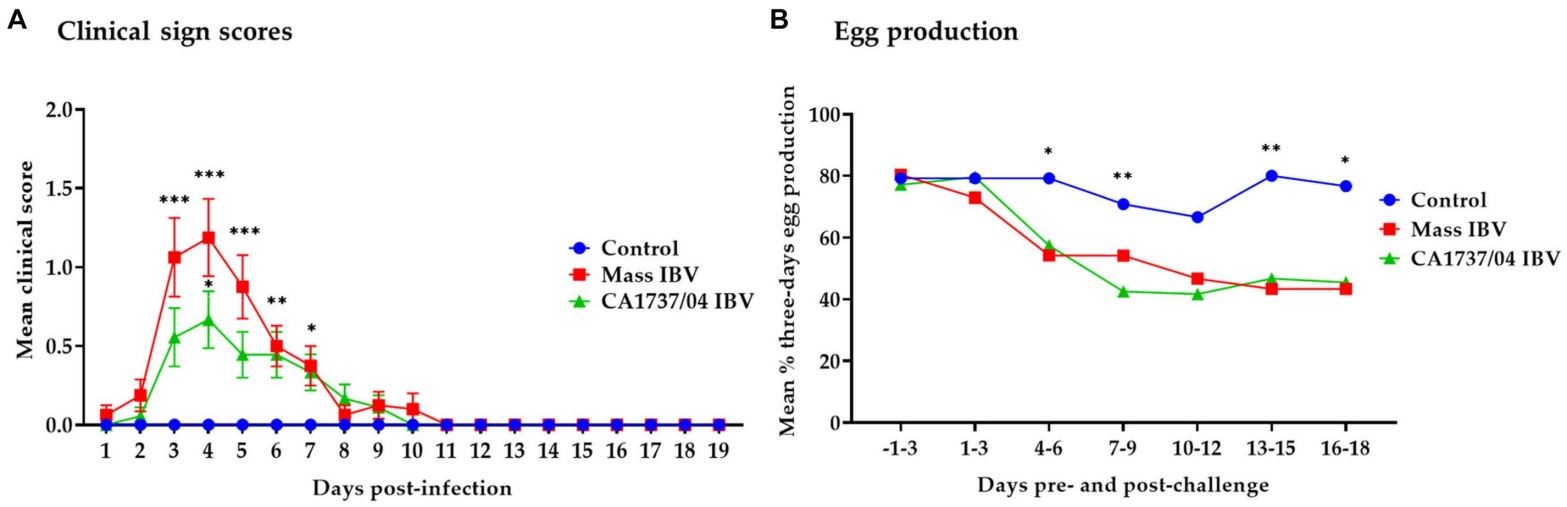
Daily mean clinical signs scores **(A)** and mean percentages of 3 days interval of egg production **(B)** following infection with Mass (15AB-01) and CA1737/04 IBV strains. The group differences in the mean clinical signs scores were identified by the Kruskal–Wallis test followed by Dunn’s multiple comparison test. Error bars represent the standard error of the mean (SEM). Fisher’s exact test was employed to compare the proportion of 3 days intervals of egg production. Asterisks indicate significant differences (^*^p < 0.05, ^**^p < 0.01, and ^***^p < 0.001).

The control group showed no clinical signs throughout the observation period. On the other hand, hens in the Mass and CA1737/04 IBV-infected groups demonstrated respiratory distress, including sneezing, tracheal rales, and gasping, as well as non-specific signs such as depression and ruffled feathers. These signs were observed as early as 1 and 2 dpi. The mean clinical scores of the Mass IBV-infected group were significantly higher than that calculated in the control group (Figure 1A; *p* < 0.05) from 3 to 7 dpi. In the CA1737/04 IBV-infected group, the mean clinical sign scores were significantly higher than those in the control group (*p* < 0.05) at 4 and 6 dpi. The peak of clinical signs was determined at 4 dpi in both IBV-infected groups (Figure 1A). Although the mean clinical scores in the Mass IBV-infected group were higher than those observed in the CA1737/04 IBV-infected group, no statistically significant differences could be detected between the two groups (*p* > 0.05). On 9 to 10 dpi, most birds in the Mass and CA1737/04 IBV-infected groups recovered.

The egg production of all groups from 3 days pre-infection until 18 dpi is presented in Figure 1B. In the Mass IBV-infected group, the egg production dropped to approximately 44% by 5 dpi, whereas in the CA1737/04 group, it declined to about 28% by 6 dpi. Comparing egg production during the pre-infection period to the 18 dpi, the Mass and CA1737/04 IBV-infected groups experienced average drops of 28% and 25%, respectively. While the egg production in the control group showed a slight drop of approximately 4% on average. The egg production in the Mass and CA1737/04 IBV-infected groups was significantly lower than that observed in the control group (*p* < 0.05) on days 4–6, 13–15, and 16–18 dpi. However, only the CA1737/04 IBV-infected group had a significantly lower egg production compared to the control group (*p* < 0.05) at 7–9 dpi. No significant differences in egg production between the Mass and CA1737/04 IBV-infected groups (*p* > 0.05) were detected throughout the experiment.

Both IBV strains induced changes in external and internal egg quality 3 dpi onwards. Regarding the external egg quality issues, the Mass IBV-infected hens displayed one shell-less egg and 10 eggs with rough shells. While the CA1737/04 IBV-infected hens showed only two eggs with hair cracks on the shell. In terms of the internal egg quality abnormalities, the Mass IBV-infected hens revealed five eggs with watery albumen and 3 eggs with blood spots on the yolk. On the other hand, the CA1737/04 IBV-infected hens demonstrated three eggs with watery albumen and two eggs with blood on the yolk.

### 3.2 Gross pathological lesions and oviduct length

**Figure 2:**
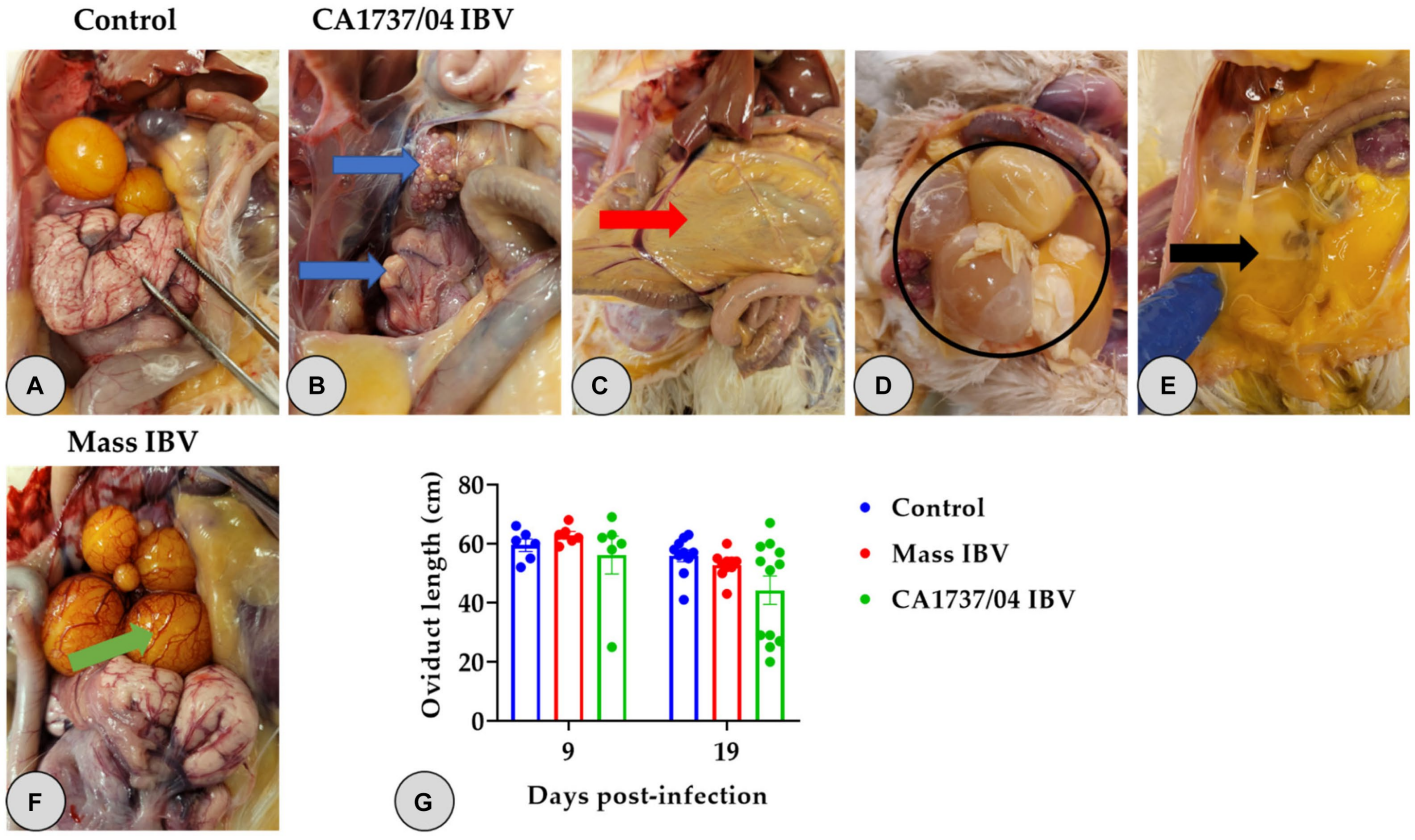
Reproductive gross lesions **(A–F)** and oviduct lengths **(G)** at 9 and 19 days following infection with Mass (15AB-01) and CA1737/04 IBV strains. **(A)** Refers to the control group without gross lesions. **(B–E)** Shows gross lesions observed in the CA1737/04 IBV-infected group; blue arrows reveal regressed ovary and oviduct; red arrow indicates yellowish peritoneum; black circle and arrow refer to eggs and ruptured yolk inside the abdominal cavity, respectively. **(F)** Shows congested ovarian follicles in the Mass IBV-infected group (green arrow). **(G)** Represents oviduct lengths means which were compared between the groups using the Kruskal–Wallis test followed by Dunn’s multiple comparison test. Error bars represent SEM.

**Figure 3:**
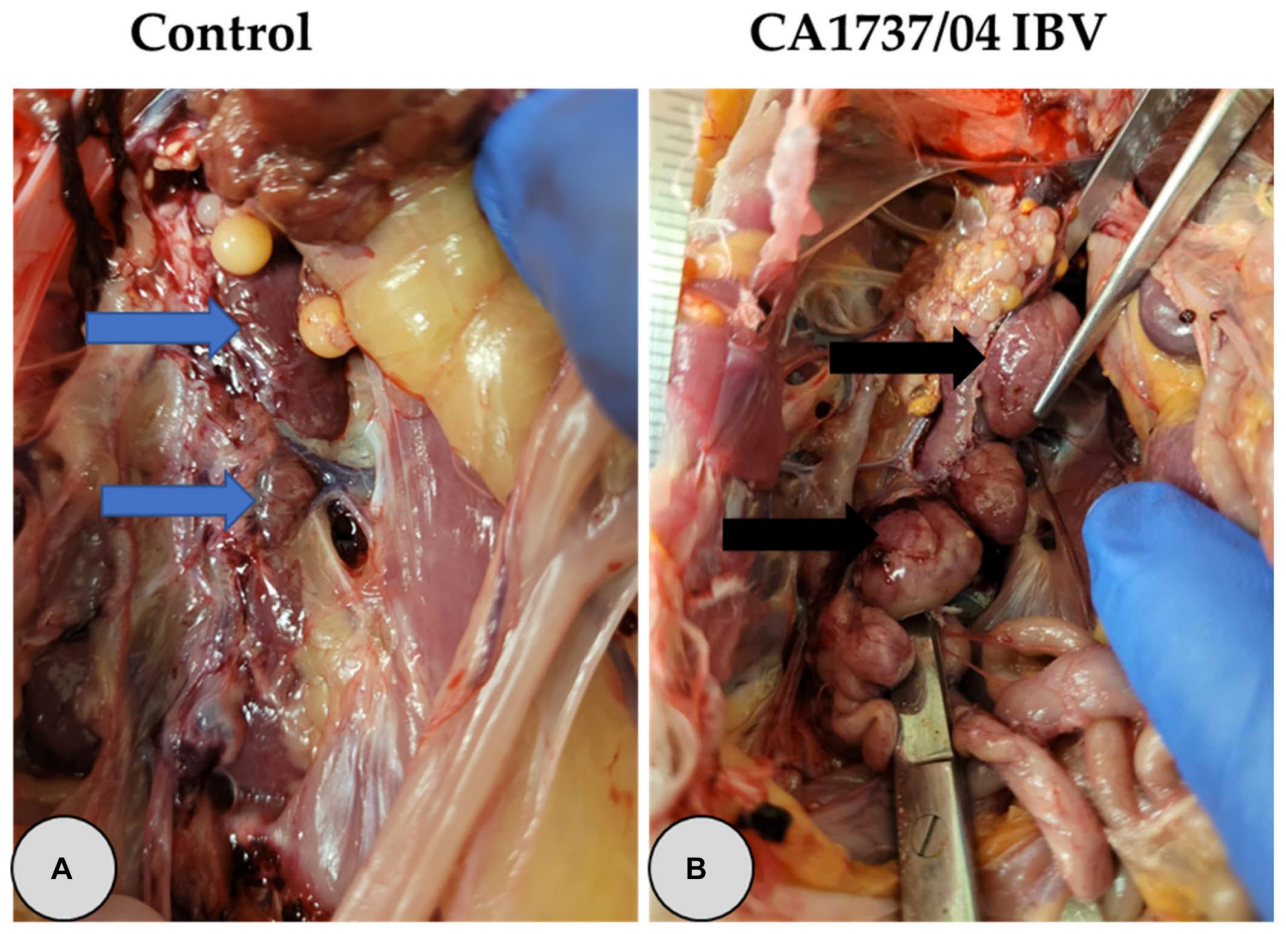
Representative gross lesions observed in the kidney at 9 and 19 days following infection with CA1737/04 IBV strain. **(A)** Refers to the control group without gross lesions (indicated with blue arrows). **(B)** Reveals swollen kidney (shown by black arrows).

No gross lesions were inspected in the control group at 9 and 19 dpi (Figures 2A, 3A). However, different lesions were observed in the reproductive system of infected hens (Table 2). In CA1737/04 IBV-infected group, the reproductive gross lesions were detected in both the ovary and oviduct, representing (3/6) 50% and (8/12) 66.7% out of the total infected hens at 9 and 19 dpi (Table 2), respectively. The observed gross lesions in the CA1737/04 IBV-infected group were mainly in the form of egg peritonitis and regression of both ovary and oviduct with inspissated materials inside (Figure 2B). The egg peritonitis appeared as thickened and yellowish peritoneum, and it was assumed to be caused by falling of eggs or mature follicles with subsequent rupture of yolk into the abdominal cavity (Figures 2C–E). On the other hand, the gross lesions in the Mass IBV-infected group were primarily restricted to the ovary, representing 16.7% (1/6) and 10% (1/10) out of the total infected females at 9 and 19 dpi, respectively (Table 2). The infected females exhibited inflamed and congested ovarian follicles (Figure 2F).

**Table 2:** Necropsy findings of the reproductive organs at 9 and 19 days following infection with Mass IBV (15AB-01) and CA1737/04 IBV strains.

Lesions	9 dpi			19 dpi		
	Control	Mass IBV	CA1737/04 IBV	Control	Mass IBV	CA1737/04 IBV
Egg peritonitis	0/6	0/6	2/6	0/10	0/10	3/12
Regressed oviduct and ovary	0/6	0/6	1/6	0/10	0/10	5/12
Congested ovary	0/6	1/6	0/6	0/10	1/10	0/12
Total	0/6 (0%)	1/6 (16.7%)	3/6 (50%)	0/10 (0%)	1/10 (10%)	8/12 (66.7%)

One hen (1/6) and two hens (2/12) of the CA1737/04 IBV-infected group experienced marked swelling of the kidney with urate deposition at 9 and 19 dpi (Figure 3B), respectively. Considering oviduct length, there were no significant differences in the mean of oviduct length between infected and mock-infected groups (*p* > 0.05) at 9 and 19 dpi.

### 3.3 Antibody-mediated immune response

**Figure 4:**
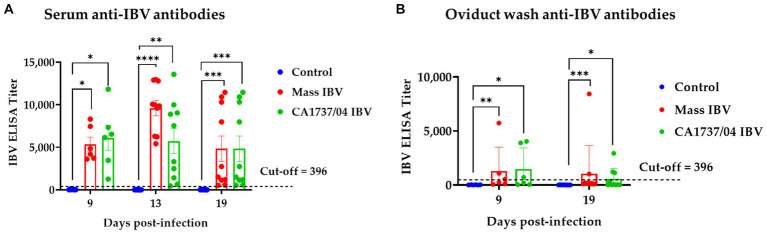
Anti-IBV antibodies in serum **(A)** and oviduct washes **(B)** collected at different time points following infection with Mass IBV (15AB-01) and CA1737/04 IBV strains. The mean titers were compared between the experimental groups using the Kruskal–Wallis test and Dunn’s multiple comparison test. Error bars represent SEM. Asterisks indicate significant differences (^*^p < 0.05, ^**^p < 0.01, ^***^p < 0.001, and ^****^p < 0.0001).

The anti-IBV antibody titers in all groups in the serum and oviduct wash are shown in Figures 4A,B, respectively. The sera and oviduct washes of the control group had no specific anti-IBV antibody titers during the experimental period. The antibody titers in sera of the Mass and CA1737/04 IBV-infected groups were significantly higher than that monitored in the control group (*p* < 0.05) at 9, 13, and 19 dpi. No significant differences in serum anti-IBV antibodies between the Mass and CA1737/04 IBV-infected groups (*p* > 0.05) were observed throughout the experiment.

Similarly, the CA1737/04 and Mass IBV-infected groups had significantly higher anti-IBV antibodies in oviduct wash than those measured in the control group (*p* < 0.05) at 9 and 19 dpi. There were no significant differences between the two infected groups (*p* > 0.05) in anti-IBV antibody titers at both time points.

### 3.4 IBV genome load quantification

#### 3.4.1 IBV genome loads in oropharyngeal and cloacal swabs

**Figure 5:**
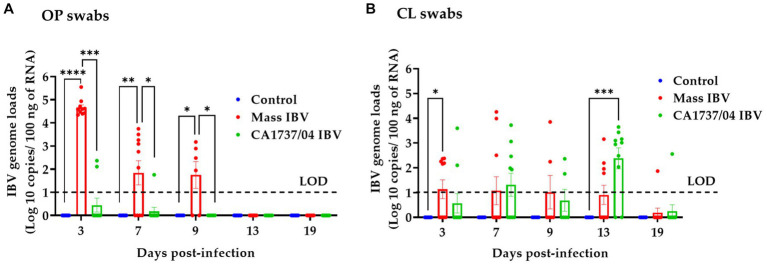
IBV genome loads were quantified from OP **(A)** and CL **(B)** swabs collected at 3, 7, 9, 13, and 19 days following infection with Mass (15AB-01) and CA1737/04 IBV strains. The Kruskal–Wallis test followed by Dunn’s multiple comparison test was used to analyze the group differences. Error bars represent SEM. Asterisks indicate significant differences (^*^p < 0.05, ^**^p < 0.01, ^***^p < 0.001, and ^****^p < 0.0001). The dotted lines indicate the limit of detection (LOD) of RT-qPCR assay.

IBV genome loads in OP and CL swabs of all groups are shown in Figures 5A,B, respectively. None of the hens in the control group demonstrated viral shedding in OP and CL swabs during the observation period. Overall, the CA1737/04 IBV-infected group showed limited IBV genome loads in OP swabs (Figure 5A). The IBV genome loads in OP swabs of the Mass group were significantly higher than that detected in the CA1737/04 IBV-infected group and the control group as well (*p* < 0.05) at 3, 7, and 9 dpi. No viral shedding was observed at 13 and 19 dpi. In terms of CL swabs, the IBV genome loads in the Mass IBV-infected group were significantly higher than those of the control group (*p* < 0.05) at 3 dpi. However, no significant difference could be detected between the groups (*p* > 0.05) at 7 and 9 dpi. IBV genome loads in the CA1737/04 IBV-infected group were significantly higher compared to the control group (*p* < 0.0.05) at 13 dpi. There was no significant difference in the IBV genome loads between all experimental groups (*p* > 0.05) at 19 dpi.

#### 3.4.2 IBV genome loads in tissues

**Figure 6:**
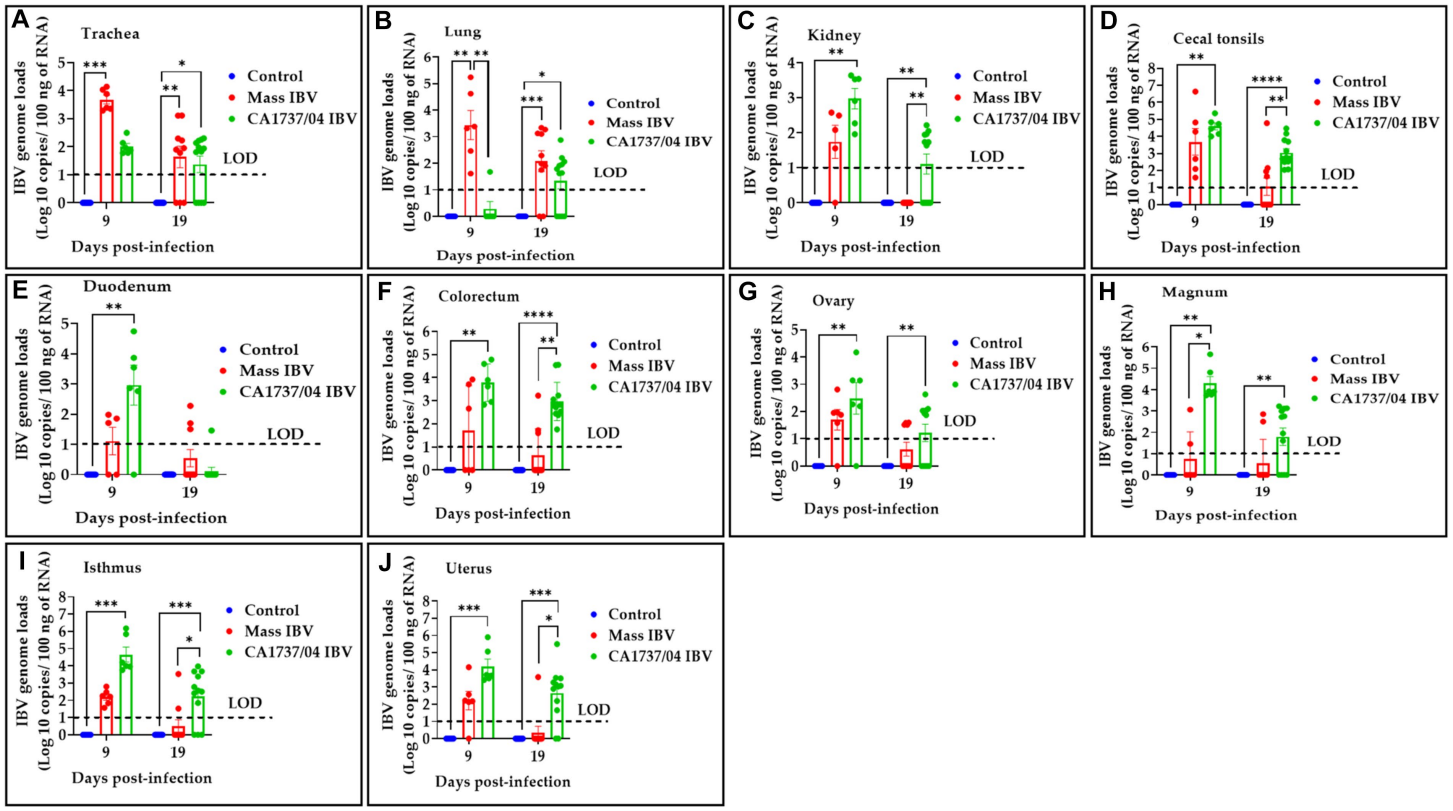
IBV genome loads were determined in trachea **(A)**, lung **(B)**, kidney **(C)**, cecal tonsils **(D)**, duodenum **(E)**, colorectum **(F)**, ovary **(G)**, magnum **(H)**, isthmus **(I)**, and uterus **(J)** collected at 9 and 19 days following infection with Mass (15AB-01) and CA1737/04 IBV strains. The group difference was identified using the Kruskal–Wallis test followed by Dunn’s multiple comparison test. Error bars represent SEM. Asterisks indicate significant differences (^*^p < 0.05, ^**^p < 0.01, ^***^p < 0.001, and ^****^p < 0.0001). The dotted lines show the limit of detection (LOD) of RT-qPCR assay.

The IBV genome loads in different tissues of all experimental groups obtained at 9 and 19 dpi are illustrated in Figure 6. No IBV genome loads were detectable in all tissues of the control group throughout the experiment. The collected tissues from the CA1737/04 and Mass IBV-infected groups showed detectable IBV genome loads; however, the significant difference between the experimental groups varied for each individual tissue and time point.

At 9 dpi, the CA1737/04 IBV-infected group had significantly higher IBV genome loads than those detected in the control group (*p* < 0.05) in all tissues except the trachea and lung (*p* > 0.05). At the same time point, the CA1737/04 IBV-infected group showed significantly higher IBV genome loads when compared to those determined in the Mass IBV-infected group in magnum (*p* < 0.05). On the other hand, the Mass IBV-infected group exhibited higher IBV genome loads than those measured in the CA1737/04 IBV-infected group and the control group (*p* < 0.05) in the lung. Furthermore, the IBV genome loads in the trachea of the Mass IBV-infected group were significantly higher than those observed in the control group (*p* < 0.05).

Continuously, at 19 dpi, the CA1737/04 IBV-infected group demonstrated higher IBV genome loads than those determined in the control group (*p* < 0.05) in all tissues except duodenum (*p* > 0.05). Among these tissues (kidney, cecal tonsils, colorectum, isthmus, and uterus), there were significantly higher IBV genome loads in the CA1737/04 IBV-infected group when compared to those detected in the Mass IBV-infected group (*p* < 0.05).

### 3.5 Histopathological findings

**Figure 7:**
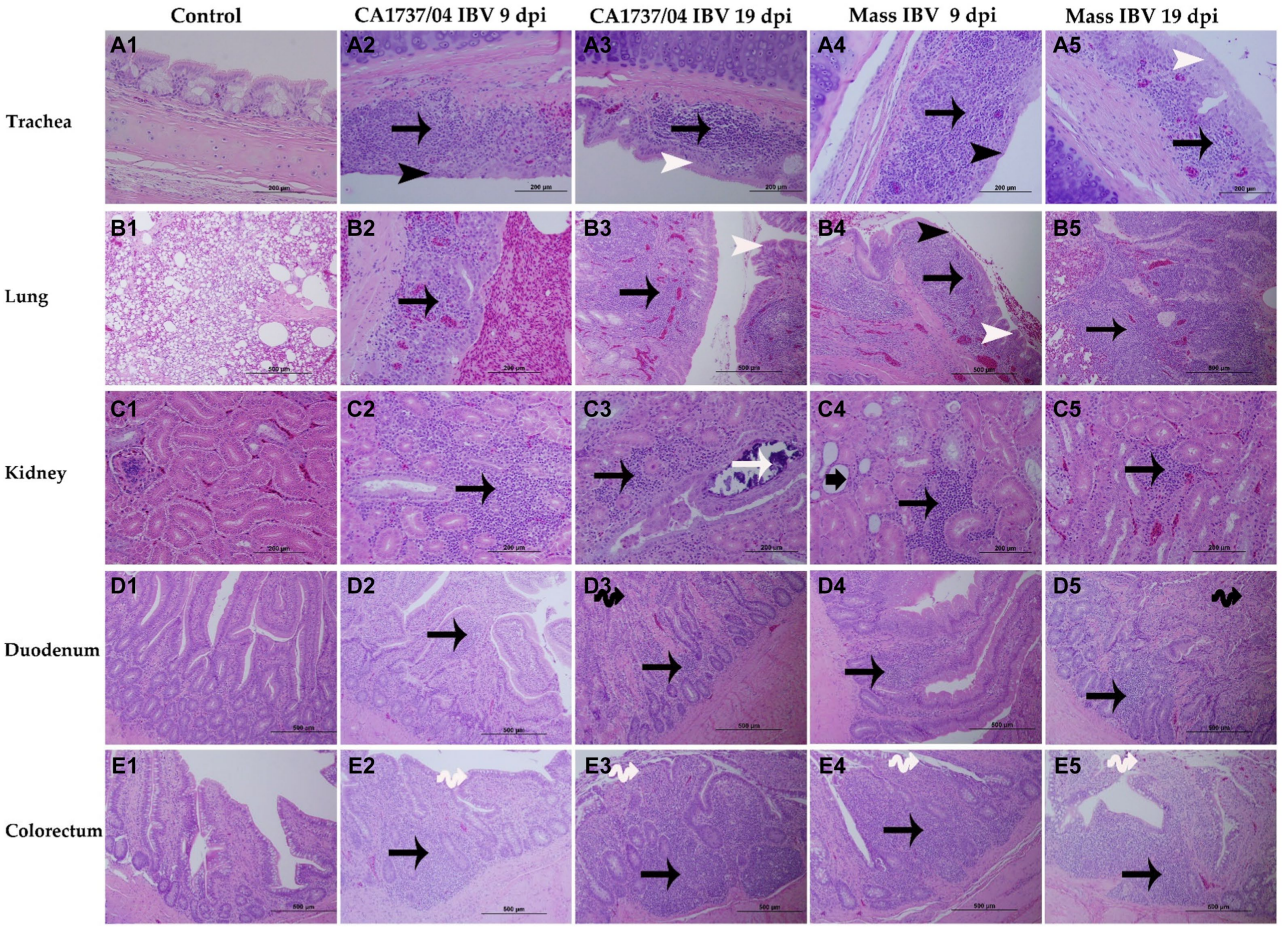
Microscopic changes observed in the trachea **(A1–A5)**, lung **(B1–B5)**, kidney **(C1–C5)**, duodenum **(D1–D5)**, and colorectum **(E1–E5)** at 9 and 19 days following infection with Mass (15AB-01) and CA1737/04 IBV strains. **A1–E1** are controls. **A2–E2** and **A3–E3** show lesions in the CA1737/04 IBV-infected group at 9 and 19 dpi, respectively. While **A4–E4** and **A5–E5** refer to lesions in the Mass IBV-infected group at 9 and 19 dpi, respectively. Black arrows indicate inflammatory cell infiltrations; black head arrows show epithelial thinning; white head arrows refer to epithelial hyperplasia; white arrow shows intra-luminal dark blue deposits; short-thick black arrow refers to tubular dilatation; double black arrows indicate villus atrophy; double white arrows show necrosis of epithelial cells from intestinal villi.

No obvious microscopic lesions were observed in any of the examined tissues of the control group throughout the study (Figures 7A1–E1, 8F1–I1). However, in both IBV-infected groups, typical IBV-induced histopathological lesions were evident in the collected tissues at 9 and 19 dpi.

**Figure 8:**
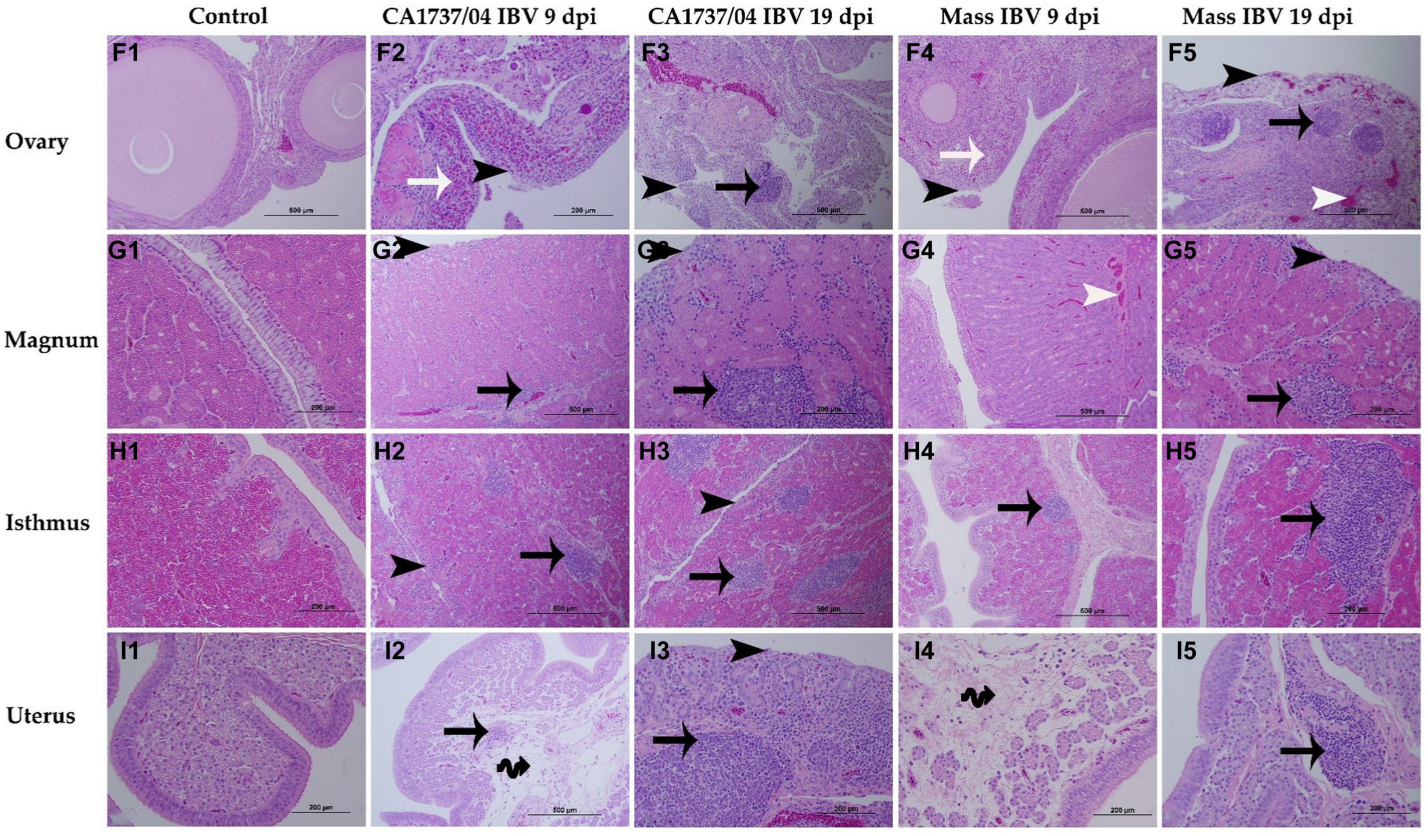
Microscopic changes observed in the ovary **(F1–F5)**, magnum **(G1–G5)**, isthmus **(H1–H5)**, and uterus **(I1–I5)** at 9 and 19 days following infection with CA1737/04 and Mass IBV strains. **F1–I1** are controls. **F2–I2** and **F3–I3** represent lesions detected in the CA1737/04 IBV-infected group at 9 and 19 dpi, respectively. Whereas, **F4–I4** and **F5–I5** show lesions observed in the Mass group at 9 and 19 dpi, respectively. Black-headed arrows refer to epithelial and ciliary losses; white-headed arrows show congested blood capillaries; black arrows indicate mononuclear cell infiltrations; white arrows refer to heterophils; double black arrows show edema.

**Figure 9:**
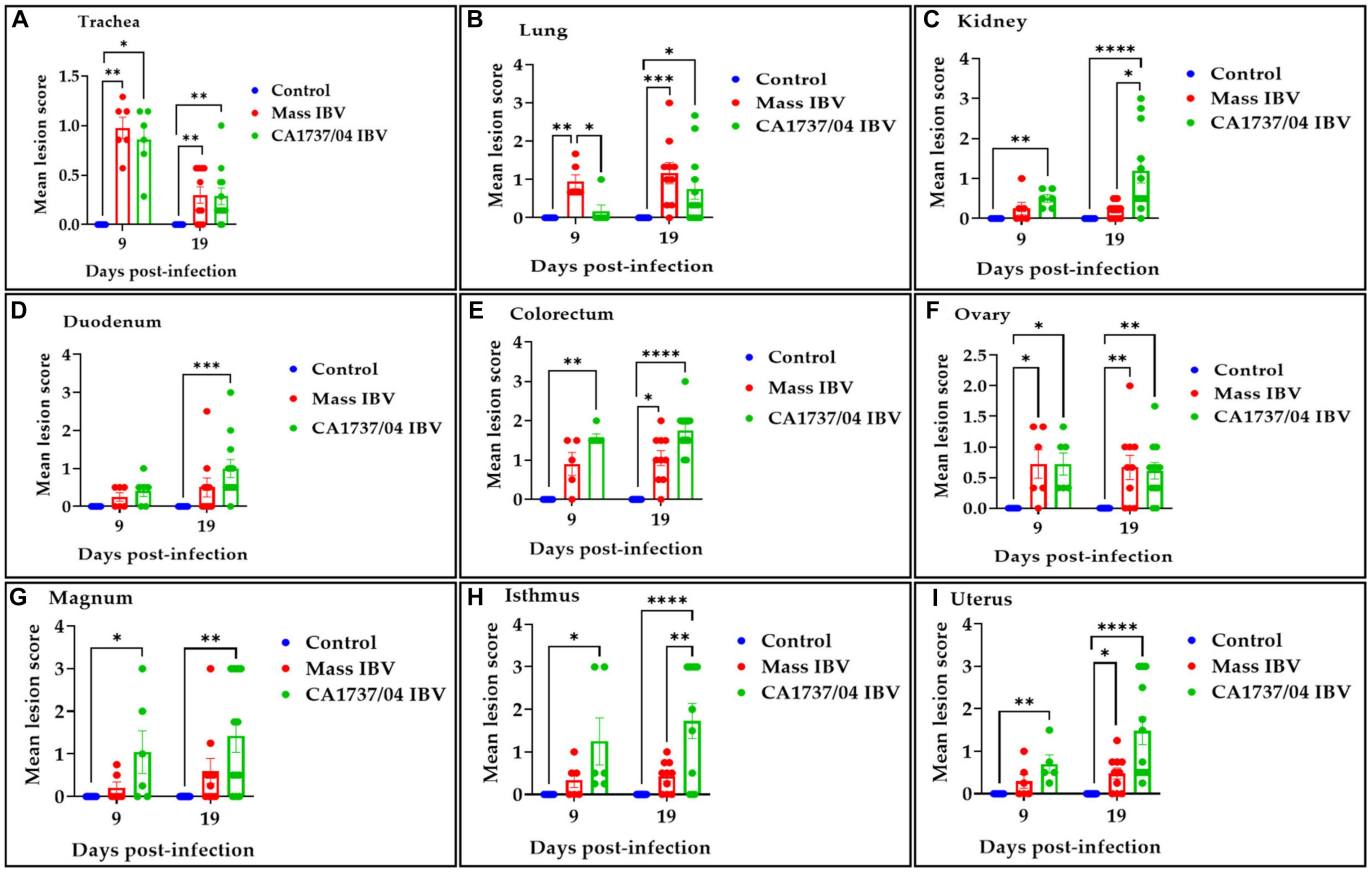
Histopathological lesions were scored in the trachea **(A)**, lung **(B)**, kidney **(C)**, duodenum **(D)**, colorectum **(E)**, ovary **(F)**, magnum **(G)**, isthmus **(H)**, and uterus **(I)** at 9 and 19 days following infection with Mass (15AB-01) and CA1737/04 IBV strains. The mean scores were compared among the groups using the Kruskal–Wallis test followed by Dunn’s multiple comparison test. Error bars represent SEM. Asterisks indicate significant differences (^*^p < 0.05, ^**^p < 0.01, ^***^p < 0.001, and ^****^p < 0.0001).

In the trachea, both IBV-infected groups showed a similar pattern of tracheal lesions. The lesions were characterized by epithelial thinning with deciliation, loss of goblet cells, and dilated mucosal glands at 9 dpi. In addition, the lamina propria was thickened with diffuse lymphoplasmacytic cell infiltrations (Figures 7A2,A4). At 19 dpi, the pathological lesions were mainly less intensive and consisted of epithelial hyperplasia and lymphoplasmacytic cell aggregations beneath the epithelium (Figures 7A3,A5). There were no significant differences between the two IBV-infected groups in regard to tracheal lesion scores (*p* > 0.05); however, the two groups had significantly higher pathological lesions than those calculated in the control group (*p* < 0.05) at 9 and 19 dpi (Figure 9A).

The lung lesions were predominantly found in the secondary bronchi. The mucosa of secondary bronchi revealed epithelial hyperplasia and/or thinning, and the subepithelial tissue was expanded due to mononuclear cell infiltrations and congested blood capillaries (Figures 7B2,B4). These lesions were seen in most hens of the Mass IBV-infected group and only one hen in the CA1737/04 IBV-infected group at 9 dpi. The bronchial lesions persisted at 19 dpi in the CA1737/04 group (Figure 7B3). However, in some hens of the Mass IBV-infected group, the lesions occupied a wide area of the lung parenchyma, in which there was marked necrosis of atria and infundibula with diffuse infiltrations of the mononuclear cells and eosinophilic proteinaceous materials (Figure 7B5). The Mass group had significantly higher lung lesion scores than those calculated in the CA1737/04 IBV-infected group and the control group (*p* < 0.05) at 9 dpi. Compared to the control group, the lesion scores in the two infected groups were significantly higher (*p* < 0.05) at 19 dpi (Figure 9B).

In the kidney, the microscopic lesions consisted of lymphoplasmacytic cell infiltrations in the interstitial tissue in most hens of the CA1737/04 IBV-infected group at 9 dpi. However, only one bird in the Mass group showed moderate interstitial lymphoplasmacytic cell infiltrations with tubular dilatation (Figures 7C2,C4). The CA1737/04 IBV-infected group showed severe lesions at 19 dpi, including epithelial necrosis with sloughing, intra-luminal dark blue deposits, and lymphoplasmacytic or heterophilic infiltrations of the interstitium (Figure 7C3). On the other hand, few hens in the Mass IBV-infected group had focally mild mononuclear cell infiltrations in the interstitial tissue at 19 dpi (Figure 7C5). The renal histopathological lesion scores were significantly higher in the CA1737/04 IBV-infected group compared to the control group at 9 dpi (Figure 9C; *p* < 0.05). At 19 dpi, the CA1737/04 IBV-infected group had significantly higher lesion scores than those observed in the Mass IBV-infected group and the control group (*p* < 0.05).

As for enteric tissues, the microscopic changes in duodenum included focal infiltrations of mononuclear cells, occasionally heterophils in the lamina propria at 9 dpi; however, few hens had atrophied villi at 19 dpi in both infected groups (Figures 7D2–D5). In the colorectum, there were degeneration and necrosis with occasional sloughing of epithelial cells of the intestinal villi, and the subepithelial tissue was diffusely or focally infiltrated with lymphohistiocytic cells at 9 dpi. These lesions were continuous until 19 dpi in the two infected groups (Figures 7E2–E5). The lesion scores in the colorectum of the CA1737/04 IBV-infected group were significantly more severe than those observed in the control group (*p* < 0.05) at 9 dpi. At 19 dpi, the lesion scores of the colorectum of both IBV-infected groups were significantly higher compared to the control group (Figure 9E; *p* < 0.05). In terms of the duodenum, the lesion scores in the CA1737/04 IBV-infected group were significantly higher than those calculated in the control group at 19 dpi (Figure 9D; *p* < 0.05).

In the ovary, the histopathological lesions were almost identical in both CA1737/04 and Mass IBV-infected groups. These lesions consisted of patchy necrosis of ovarian epithelium, and the cortical stroma was infiltrated focally or multi-focally with inflammatory cell infiltrations mainly of heterophils and/or mononuclear cells-types, in addition to congestion of blood capillaries (Figures 8F2–F5). Both IBV-infected groups had significantly severe pathological lesion scores compared to those observed in the control group (*p* < 0.05), however, no significant difference could be detected between them (Figure 9F; *p* > 0.05) at 9 and 19 dpi.

In different portions of the oviduct (magnum, isthmus, and uterus), the histopathological lesions were more evident in the CA1737/04 IBV-infected group than in the Mass IBV-infected group. In the CA1737/04 IBV-infected group, the lesions of magnum and isthmus consisted of epithelial and ciliary losses, mononuclear cell infiltrations, edema, and congestion of blood capillaries in lamina propria at 9 dpi (Figures 8G2,H2). These lesions became more expanded and pronounced at 19 dpi (Figures 8G3,H3). In the uterus of the CA1737/04 IBV-infected group, there were intact ciliated epithelium, few mononuclear cell infiltrations, and edema in lamina propria at 9 dpi (Figure 8I2). However, the epithelia and cilia were lost together with the expansion of inflammatory cell infiltration beneath the epithelium at 19 dpi (Figure 8I3). On the other hand, the microscopic findings in the Mass IBV-infected group were in the form of intact epithelium and thickened lamina propria as a result of edema and infiltrations of mononuclear cells, particularly in the isthmus and uterus (Figures 8H4,H5,I4,I5). In the magnum, the lesions were only characterized by congested blood capillaries beneath the epithelium at 9 dpi (Figure 8G4). However, few hens exhibited epithelial sloughing, deciliation, and infiltrations of mononuclear cells in lamina propria at 19 dpi (Figure 8G5). The lesion scores in the CA1737/04 IBV-infected group were significantly higher than those calculated in the control group (*p* < 0.05) at 9 and 19 dpi. Furthermore, the CA1737/04 IBV-infected group had significantly higher histopathological lesion scores than those calculated in the uterus of the Mass IBV-infected group (Figures 9G–I; *p* < 0.05).

### 3.6 Immunohistochemistry

**Figure 10:**
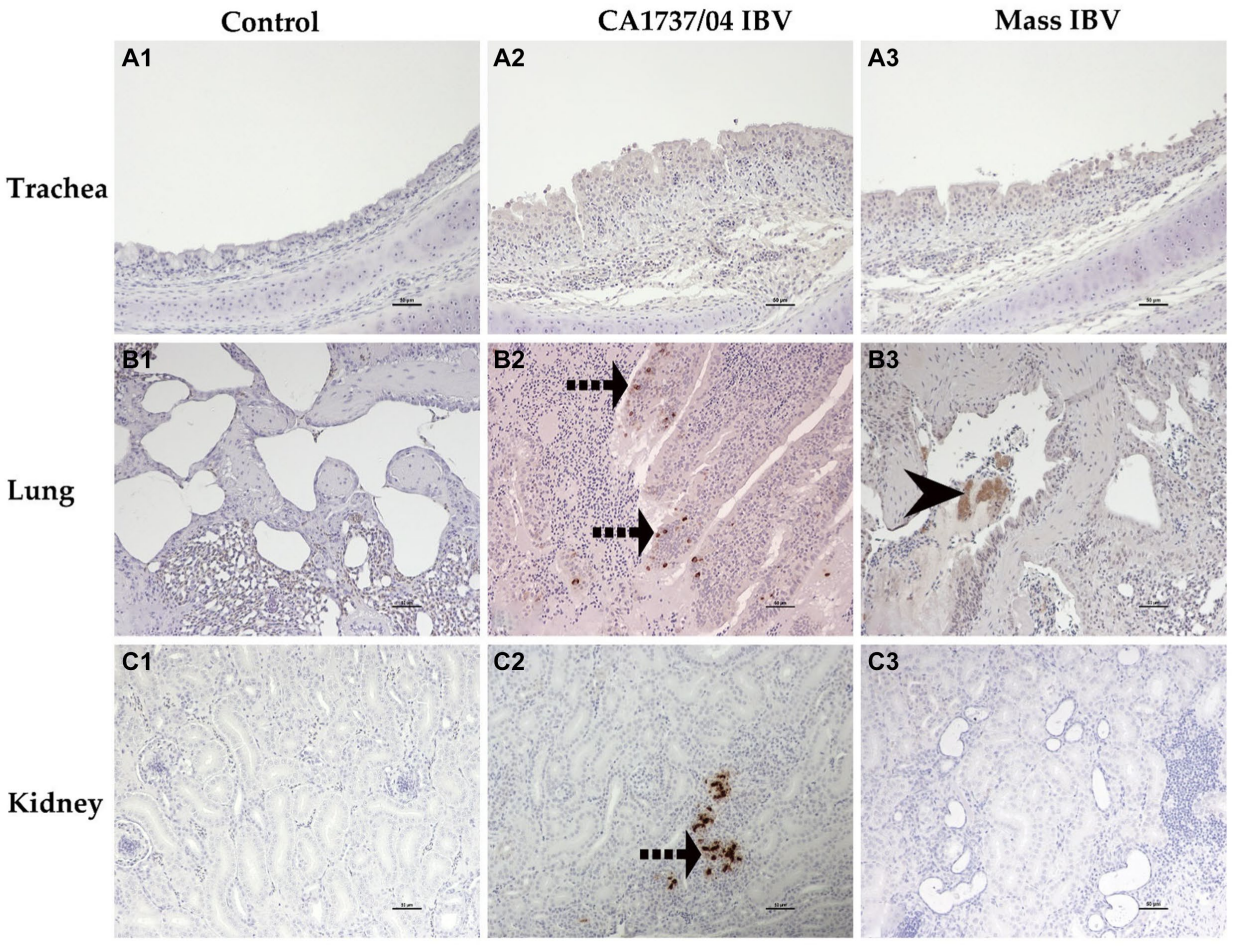
Immunohistochemical detection of IBV antigen in trachea **(A1–A3)**, lung **(B1–B3)**, and kidney **(C1–C3)** collected at 9 and 19 dpi. **A1–C1** are controls. **A2,A3** represent no expression of IBV antigen in trachea of both IBV-infected groups. **B2,C2** reveal expression of IBV antigen in the CA1737/04-IBV infected group. **B3** shows expression of IBV antigen in the Mass IBV-infected group. Dotted black arrows and black head arrow indicate viral antigen immunolabeling in intact and sloughed epithelia, respectively. Scale bar = 50 μm.

The IHC assay was used to determine the viral antigen distribution in the trachea, lung, kidney, cecal tonsils, duodenum, colorectum, ovary, and different parts of the oviduct at 9 and 19 dpi. The examined tissues of the control group showed no immunoreactivity throughout the experiment (Figures 10A1–C1, 11D1–F1, 12G1–J1).

**Figure 11:**
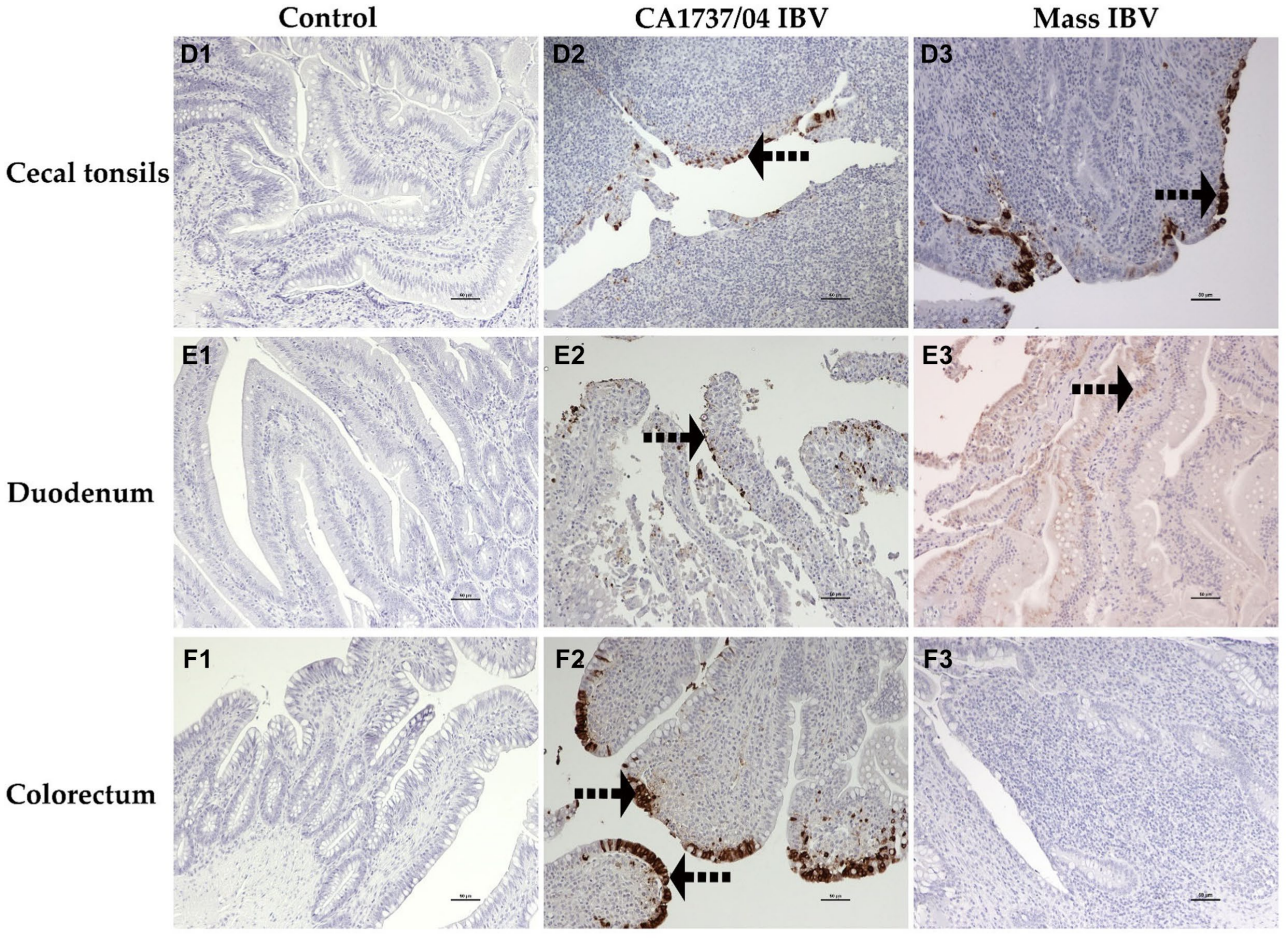
Immunohistochemical detection of IBV antigen in cecal tonsils **(D1–D3)**, duodenum **(E1–E3)**, and colorectum **(F1–F3)** collected at 9 and 19 dpi. **D1–F1** are controls. **D2–F2** represents the expression of IBV antigen in the CA1737/04 IBV-infected group. **D3,E3** show expression of IBV antigen in the Mass-infected group. The dotted black arrows refer to viral antigen immunolabeling in the epithelial cells. Scale bar = 50 μm.

**Figure 12:**
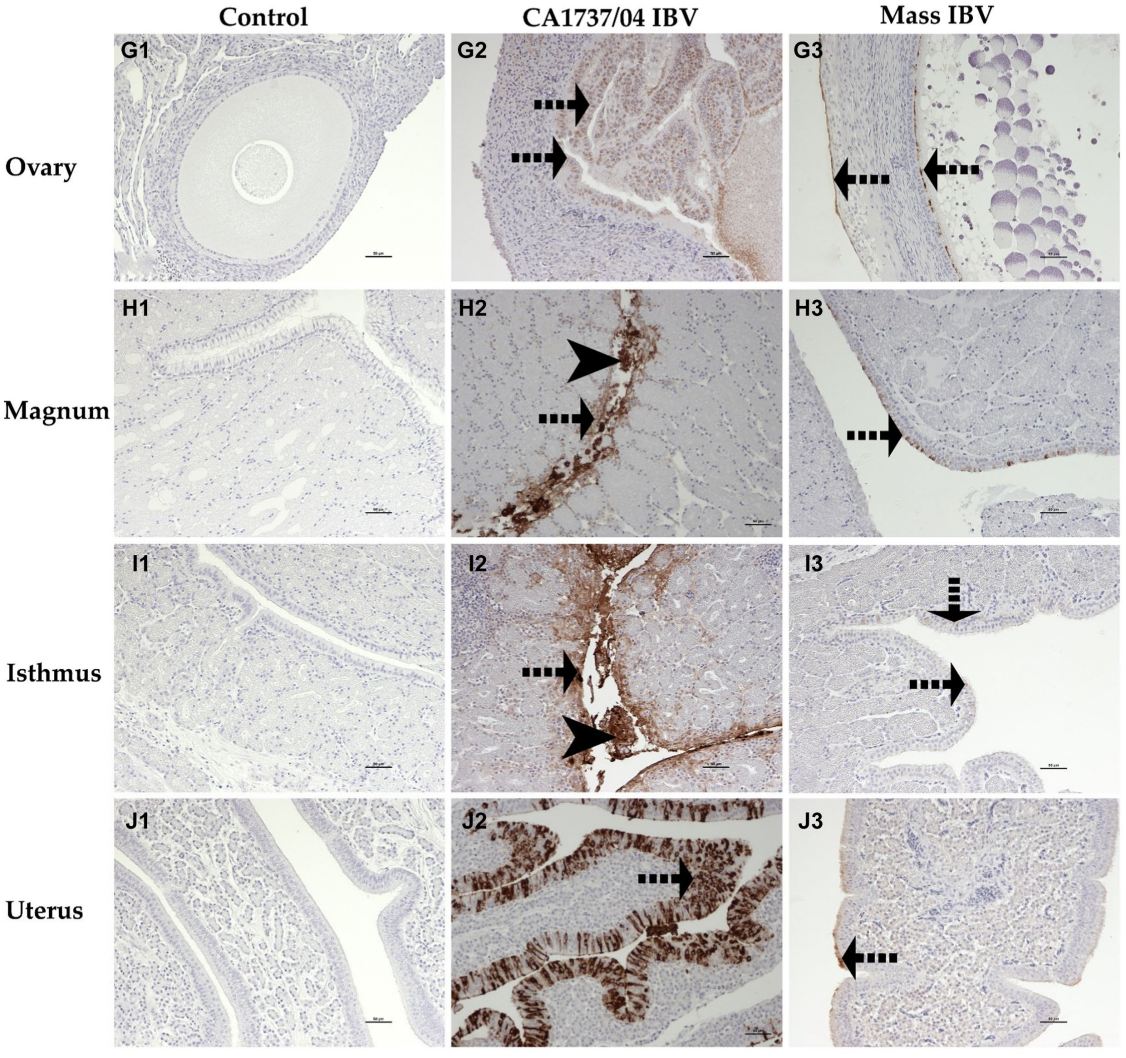
Immunohistochemical detection of IBV antigen in the ovary **(G1–G3)**, magnum **(H1–H3)**, isthmus **(I1–I3)**, and uterus **(J1–J3)** collected at 9 and 19 dpi. **G1–J1** are controls. **G2–J2** represent expression of IBV antigen in the CA1737/04 IBV-infected group. **G3–J3** show expression of IBV antigen in the Mass IBV-infected group. Dotted black arrows and black head arrows indicate viral antigen immunolabeling in intact and sloughed epithelia, respectively. Scale bar = 50 μm.

In the CA1737/04 IBV-infected group, all tested tissues had positive immunolabeling except the trachea. In contrast, only the lung, cecal tonsils, duodenum, ovary, and isthmus had positive immunostaining in the Mass IBV-infected group at the same time point. At 19 dpi, the CA1737/04 IBV-infected group showed persistent immunoreactivity in most tissues except the lung and duodenum. However, immunostaining in the Mass IBV-infected group was limited to the ovary and various oviduct segments.

The expression of viral antigen was mostly confined to the epithelial cells of different tissues in both infected groups. In the trachea, both CA1737/04 and Mass IBV strains did not exhibit any immunoreactivity (Figures 10A2,A3). While, in the lung, the immunopositivity was limited to the epithelial cells lining the secondary bronchi. The positive epithelia were either intact in the CA1737/04 IBV-infected group or exfoliated inside the lumen in the Mass IBV-infected group (Figures 10B2,B3). While in the kidney, the antigen-positive cells in the CA1737/04 IBV-infected group were detected in the cytoplasm of tubular epithelial cells (Figure 10C2). However, the kidney of the Mass IBV-infected group revealed no immunoreactivity throughout the study (Figure 10C3). In the cecal tonsils and duodenum of both IBV-infected groups, the viral antigen was expressed in the cytoplasm of intestinal epithelial cells throughout the villi (Figures 11D2,D3,E2,E3). In the colorectum of the CA1737/04 IBV-infected group, the positive cells were mostly observed in the cytoplasm of enterocytes at the tips of the intestinal villi (Figure 11F2). In contrast, the colorectum in the Mass-IBV-infected group showed no immunostaining (Figure 11F3). In the ovary, the viral antigen in both IBV-infected groups was localized in the ovarian covering epithelium and/or the granulosa cells lining the growing follicles (Figures 12G2,G3). In the magnum, isthmus, and uterus, the CA1737/04 IBV-infected group had stronger immunoreactivity than that of the Mass IBV-infected group. The immunolabeling was primarily observed in the epithelial cells which were either intact to the mucosa or sloughed inside the lumen particularly in the CA1737/04 IBV-infected group (Figures 12H2,H3,I2,I3,J2,J3).

**Figure 13:**
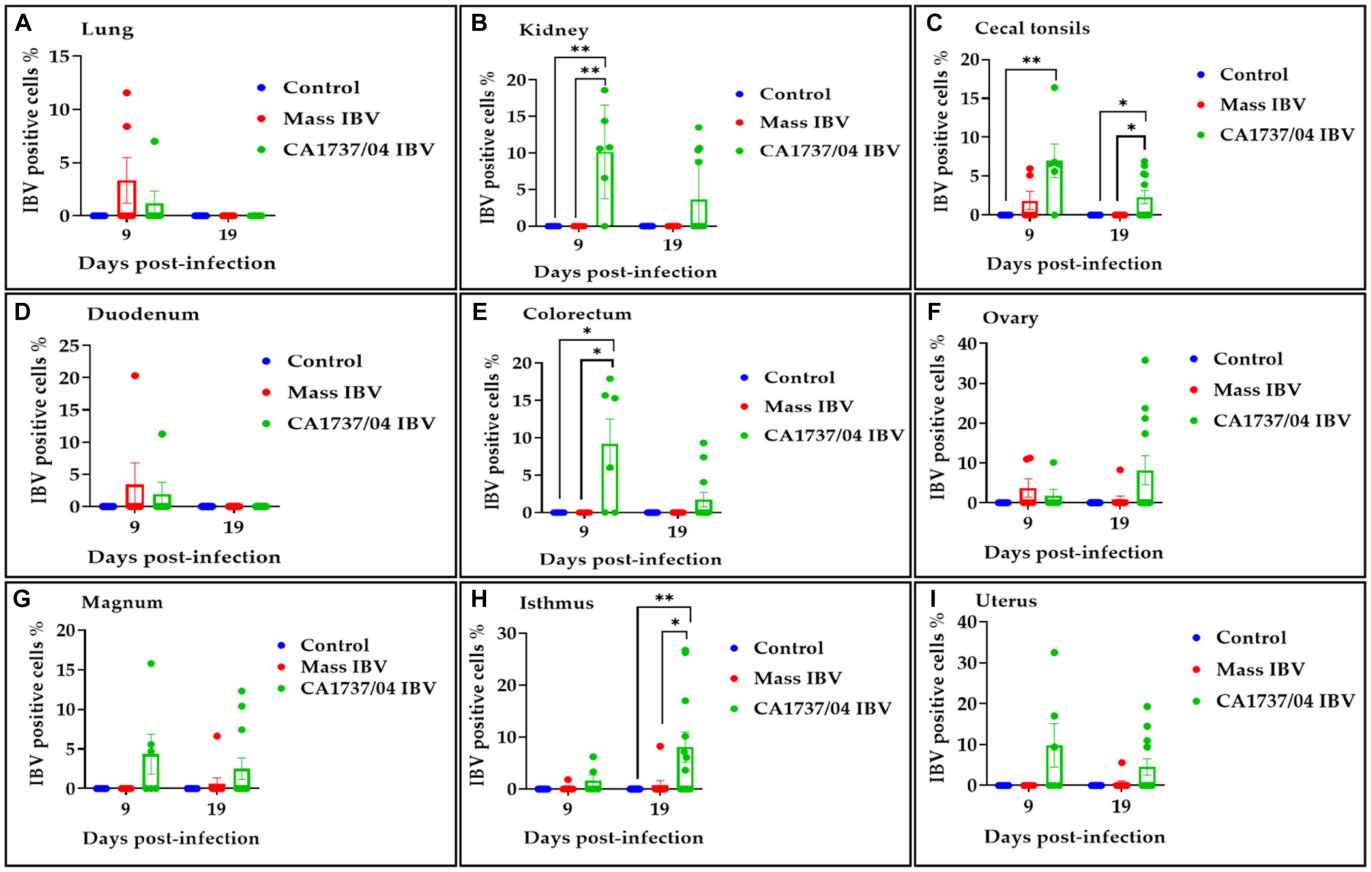
Percentage of IBV-positive cells detected in the lung **(A)**, kidney **(B)**, cecal tonsils **(C)**, duodenum **(D)**, colorectum **(E)**, ovary **(F)**, magnum **(G)**, isthmus **(H)**, and uterus **(I)** at 9 and 19 days following infection with CA1737/04 and Mass IBV strains. The Kruskal–Wallis test followed by Dunn’s multiple comparison test was used to identify the group differences. Error bars represent SEM. Asterisks indicate significant differences (^*^p < 0.05 and ^**^p < 0.01).

The IBV positive cell percentage in the tested groups is shown in Figure 13. The CA1737/04 IBV-infected group had a significantly higher percentage of IBV-positive cells than those detected in the kidney and colorectum of the Mass IBV-infected group and the control group (*p* < 0.05; Figures 13B,E). Furthermore, the percentage of positive cells in the cecal tonsils of the CA1737/04 IBV-infected group was significantly higher than those of the control group (*p* < 0.05; Figure 13C). At 19 dpi, the CA1737/04 IBV-infected group had a signficantly higher percentage of IBV-positive cells than those of the Mass IBV-infected group and the control group (*p* < 0.05; Figures 13C,H) in the isthmus and cecal tonsils.

## 4 Discussion

IBV induces pathological outcomes in multiple body systems of chickens, and it severely affects poultry production all over the world including Canada. In 2012, a new IBV strain of US origin was isolated from Ontario, Canada, called CA1737/04 IBV (28). All previous studies on CA1737/04 IBV were primarily based on field samples, in which CA1737/04 IBV was associated with respiratory disease in broiler and kidney damage in pullets (30), as well as false layer syndrome in commercial layer flocks (31). Therefore, the current study was conducted in SPF laying hens during their peak of lay to investigate the pathogenicity and tissue tropism of CA1737/04 IBV in comparison with the well-known IBV strain (Mass) (3). Our results are two-fold. First, infection of SPF laying hens with CA1737/04 or Mass IBV strain resulted in clinical signs, a significant decline in egg production, and specific anti-IBV antibodies systemically in the serum and locally in the reproductive tract wash. Second, the CA1737/04 IBV strain is more pathogenic to the kidney, colorectum (large intestine), and reproductive tract than the Mass IBV strain. The reason for this is that CA1737/04 IBV-infected tissues (kidney, colorectum, reproductive tract) showed substantial increases in one or more of the following pathogenicity parameters: gross lesions, IBV genome loads, histopathological lesion scores, and antigen localization by IHC, as compared with Mass strain.

In this context, both CA1737/04 and Mass IBV strains induced respiratory distress including sneezing, tracheal rales, and gasping, as well as non-respiratory signs such as depression and ruffled feathers. The clinical signs started as early as 1 to 2 dpi and subsided at 10 dpi; similar observations were recorded among laying chickens infected with the IBV D1466 strain (43). In terms of egg production, the onset of a drop in egg production in both CA1737/04 and Mass IBV strains was recorded within the first-week post-infection; this was in line with previous studies (14, 44, 45). The exact mechanism by which IBV induces a drop in egg production is still unclear. The adverse effects of IBV on egg production could be a result of either pathological lesions in the reproductive organs (direct effect) or indirect effects through impairing the heath status of the bird. Our findings showed that the decline in egg production in IBV-infected groups was accompanied by marked gross and histopathological lesions in the oviduct and ovary, particularly in the CA1737/04 IBV-infected group. Thereby, these lesions in the oviduct and ovary may be a factor in the decline in egg production (6). The indirect impact of IBV on egg production could be explained by decreasing the feed and water intake, which in turn adversely affects the health of the reproductive tract. Similarly, Sevoian and Levine (44) described such affected chickens after IBV infection and after withholding all food and water for 4 days. However, we did not confirm whether reduced feed and water consumption contributed to the decline in egg production.

Apart from poor egg quality, IBV could result in damage to the oviduct, disrupting normal egg development, and leading to egg quality issues such as misshaped eggs, soft-shelled eggs, and eggs with watery albumen (46). This is consistent with the current observation in which external and internal egg quality abnormalities were observed in both IBV-infected groups including shell-less eggs, eggs with hair cracks on the shell, watery albumen, and internal blood.

At necropsy, the kidneys from a few hens of the CA1737/04 IBV-infected group showed clear gross lesions, which were in the form of swollen kidneys with urates in the ureters. However, the Mass IBV-infected hens did not show any gross lesions in the kidneys. The results of histopathology in the kidneys were consistent with the gross lesions, in which the CA1737/04 IBV strain caused significantly higher histopathological lesions in the kidneys compared to those detected in the Mass IBV strain. The microscopic changes in the CA1737/04 IBV strain were predominantly interstitial lymphoplasmacytic inflammation, but heterophils were also observed. These lesions persisted throughout the experimental period (19 dpi), which may be considered as an outcome of chronic interstitial nephritis. Chronic active (ongoing) is a type of chronic interstitial nephritis and is characterized by interstitial lymphoplasmacytic inflammation with evidence of necrotic changes among the renal tubules (47). In accordance, our findings revealed the existence of necrosis of renal tubular epithelium accompanied by lymphoplasmacytic infiltrations in the interstitial tissue at 19 dpi. Taking both necropsy and histopathology results into account, we can conclude that the CA1737/04 IBV strain is more pathogenic to the kidney than the Mass IBV strain.

The gross lesions in reproductive tissues were also observed. There was a higher number of hens infected with the CA1737/04 IBV strain that had characteristic gross lesions in both the ovary and oviduct at the two euthanasia time points. While the lesions in the Mass IBV-infected group were less and restricted to the ovary. Although the histopathologic lesions were more readily observed in the oviduct of the CA1737/04 IBV-infected group than those of the Mass IBV-infected group, the pathological lesion scores did not reveal any significant difference between the two IBV strains. This could be explained by the low number of birds and the scoring system of histopathology used in this study. In the oviduct, the microscopic lesions consisted of epithelial and ciliary losses, edema, and inflammatory cell infiltrations. The later lesions were also reported in other IBV strains (13, 14, 44). In the ovary, the microscopic lesions were obvious in both CA1737/04 and Mass IBV strains, which included necrosis of ovarian covering epithelium and inflammatory cell infiltrations (mononuclear and/or heterophils). A similar pattern of ovarian lesions has been reported following infection with other IBV strains belonging to Mass and QX genotypes (48, 49).

Although the respiratory (trachea and lung) and enteric tissues (duodenum and colorectum) showed no gross lesions, there were clear microscopic changes. In trachea and lung, both CA1737/04 and Mass IBV strains had characteristic histopathological lesions typical to IBV infection; however, the viral shedding from oral swabs in the CA1737/04 IBV-infected group was significantly lower compared to the Mass group, indicating the viral shedding of the Mass strain was greater than that of the CA1737/04 IBV strain in the upper respiratory tract. In addition, the histopathological lesion scores in the lower respiratory tissue (lung) experimentally infected with the Mass IBV strain were significantly higher than those infected with the CA1737/04 IBV strain. These findings suggested that the Mass strain was associated with more severe respiratory disease than the CA1737/04 IBV strain. These findings were in line with an earlier study (31), in which another Mass IBV strain (M41) was more pathogenic to the respiratory tract than the CA1737/04 IBV strain; however, the experiment was conducted on younger chickens.

Histopathological examination of the initial part (duodenum) and terminal part (colorectum) of the intestine of both IBV-infected groups revealed increased thickness of lamina propria with mononuclear cell infiltration, and occasionally there was villus atrophy. These lesions were also observed in other IBV strains with enteropathogenicity (16, 50). The enteric lesions were associated with detectable levels of viral RNA from the cloacal swabs in both IBV-infected groups.

Regarding the humoral-mediated immune response to IBV infection, our findings showed that both IBV-infected groups had significantly higher anti-IBV antibodies in serum and oviduct washes at 9–19 dpi. In previous studies, it has been demonstrated that a significant amount of anti-IBV antibody levels was detected in oviduct washes on 7 and 23 dpi (36). Furthermore, the anti-IBV antibodies in oviduct washes originated either locally or passively from the serum (36). Apart from the role of anti-IBV antibody protection against IBV, some studies have shown a direct relationship between high levels of humoral antibodies and protection against egg production reduction in laying hens (51, 52). In addition, the use of IBV-specific antibodies in oviduct washes, rather than serum, was suggested to be a better indicator to assess egg production drop following infection (36).

In this study, IHC and qPCR assays were performed to demonstrate IBV antigen and genetic material in different tissues, respectively. Both CA1737/04 and Mass IBV-infected groups showed detectable levels of viral RNA in all tissues, including trachea, lung, kidney, cecal tonsils, duodenum, colorectum, ovary, and oviduct. A higher number of viral genome copies was found in the kidney, cecal tonsils, colorectum, and oviduct of hens inoculated with the CA1737/04 IBV strain compared with the Mass IBV strain. These results were consistent with IHC, in which the CA1737/04 strain had a significantly higher percentage of IBV-positive cells in these tissues compared to the Mass IBV strain. Unlike other tissues infected with Mass IBV strain, the lung showed higher and more persistent IBV genome loads. This may suggest that the Mass IBV strain has a restricted capability to penetrate through the blood-air barrier (in the lung) and escape the host response to spread beyond the respiratory tissues with higher genome loads, this was speculated in a previous study (53). It has been known that certain IBV strains can persist for weeks in the kidney and cecal tonsils (16, 54). Here, our findings showed that the persistence of viral RNA and antigen was not only in the kidney and cecal tonsils but also in the colorectum infected with the CA1737/04 IBV strain. It is proposed to use these organs in the identification of the CA1737/04 IBV strain in commercial chickens. Considering IHC and qPCR results, we can conclude that the CA1737/04 strain has a wider tissue tropism, including respiratory, renal, enteric, and reproductive. In addition, these results showed that the CA1737/04 IBV strain is more pathogenic to the kidney, colorectum, and oviduct than the Mass IBV strain.

To summarize, in recent years, Canadian layer flocks have encountered an increasing number of novel IBV variants of US origin, which have the potential to become the predominant IBV type. Our results revealed that the CA1737/04 IBV and Mass IBV strains were able to infect the respiratory, renal, enteric, and reproductive tissues of laying hens during their peak of laying. However, the CA1737/04 IBV strain had greater pathogenicity and tissue tropism to the kidney, large intestine (colorectum), and oviduct than the Mass IBV strain, based on one or more pathogenicity parameters. Further investigations are warranted to develop a vaccination strategy for the control of CA1737/04 IBV infection among Canadian layer flocks.

## Data availability statement

The raw data supporting the conclusions of this article will be made available by the authors, without undue reservation.

## Ethics statement

The animal study was approved by Veterinary Science Animal Care Committee (VSACC). The study was conducted in accordance with the local legislation and institutional requirements.

## Author contributions

AA: Investigation, Methodology, Validation, Writing – review & editing, Formal analysis, Writing – original draft. MF: Formal analysis, Investigation, Writing – review & editing. DA: Investigation, Writing – review & editing, Formal analysis. SN: Formal analysis, Investigation, Writing – review & editing. MH: Formal analysis, Investigation, Writing – review & editing. II: Formal analysis, Investigation, Writing – review & editing. AS: Supervision, Validation, Writing – review & editing. RG: Validation, Writing – review & editing, Conceptualization, Methodology, Resources. MA-C: Conceptualization, Methodology, Resources, Validation, Writing – review & editing, Funding acquisition, Investigation, Project administration, Software, Supervision.
