# Supplementary figures and images for: Comparative pathogenicity of CA1737/04 and Mass infectious bronchitis virus genotypes in laying chickens
Source: Front Vet Sci. 2024 Feb 28;11:1338563. doi: 10.3389/fvets.2024.1338563 (PMC10932974; doi:10.3389/fvets.2024.1338563)

**
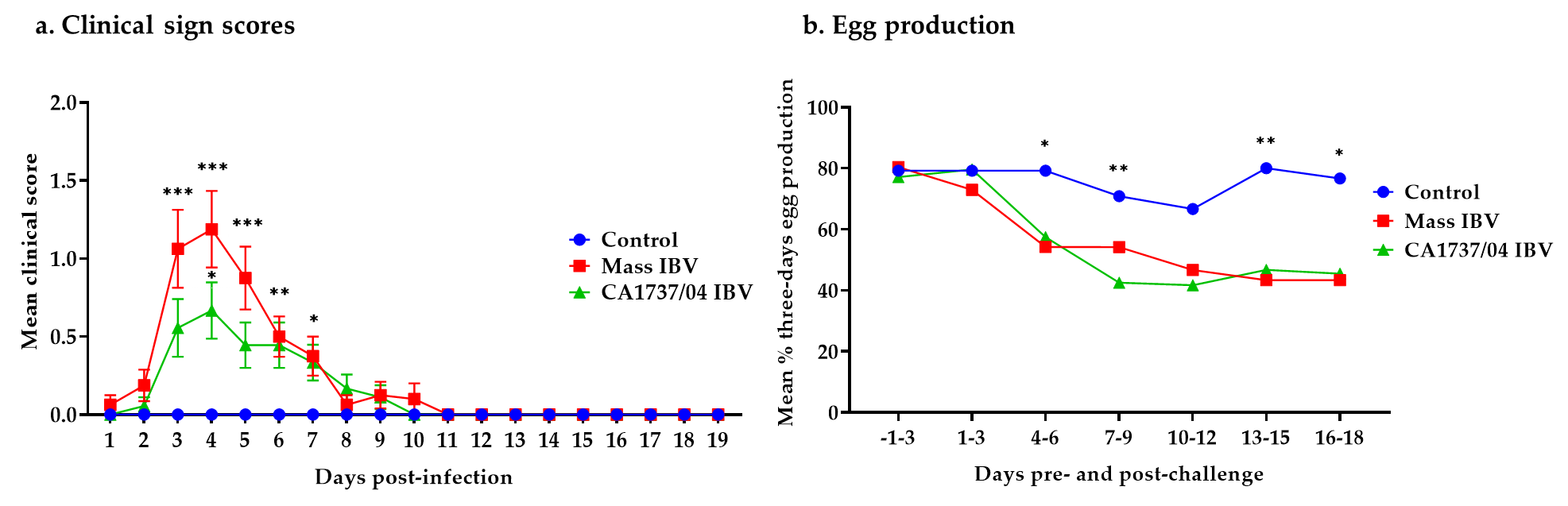
Fig. 1**

**Fig. 2**


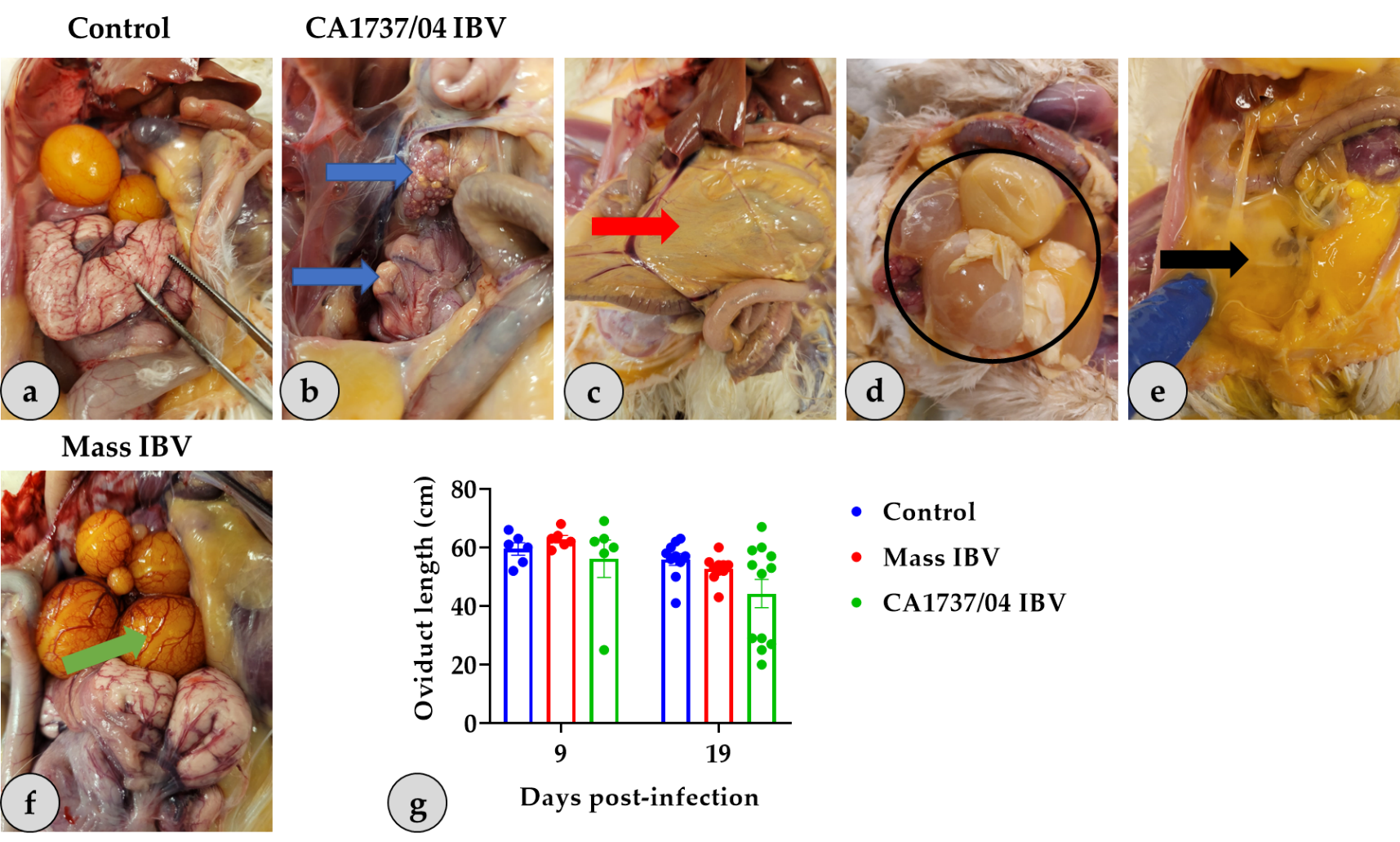


**Fig. 3**

**
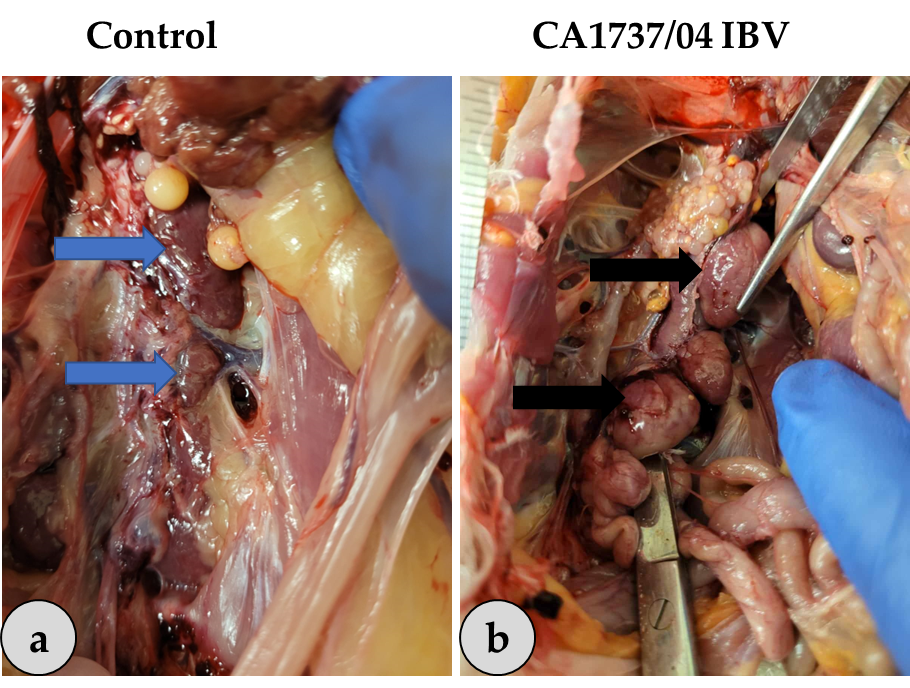
**

**
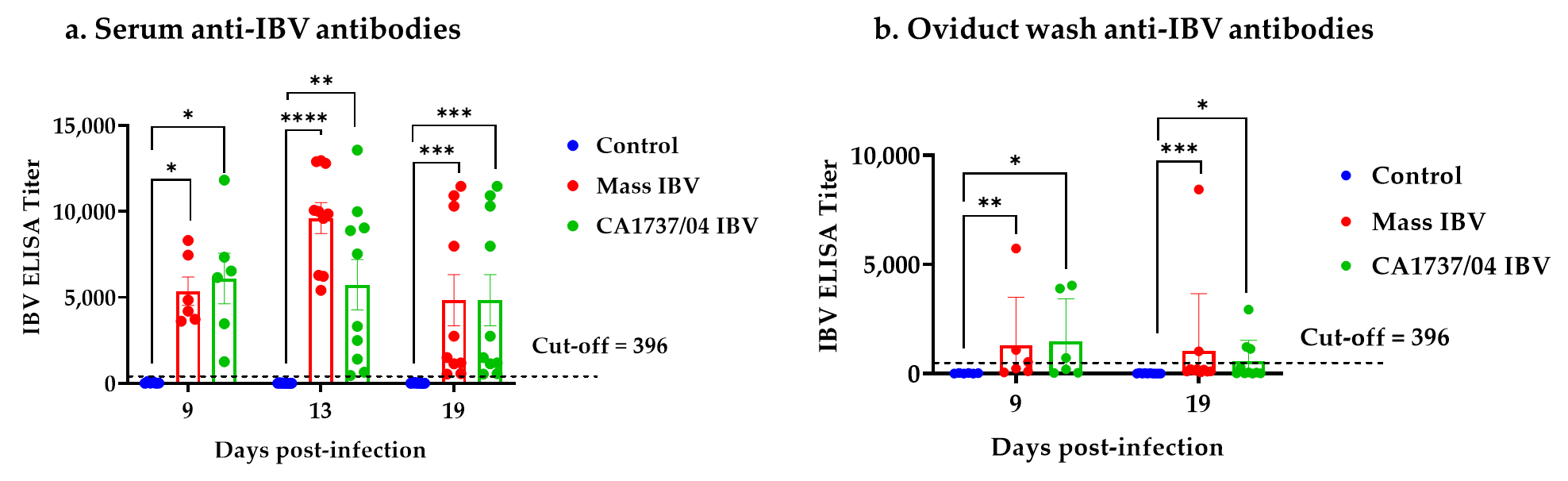
Fig. 4**

**Fig. 5**

**
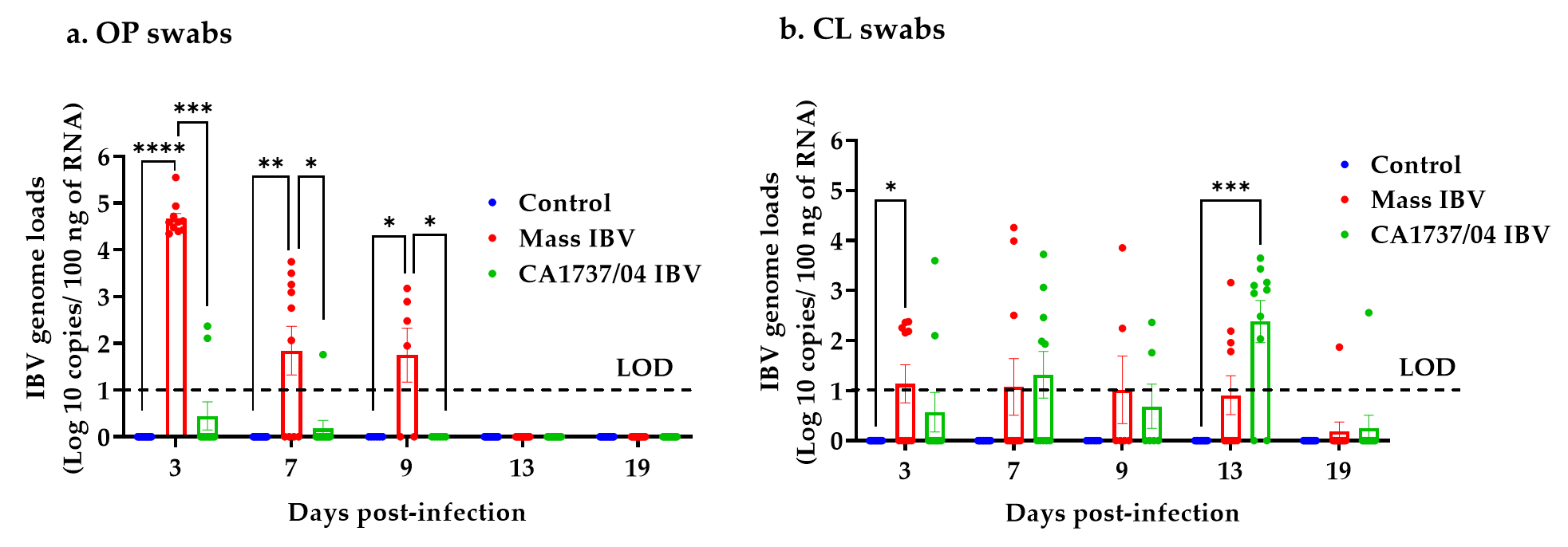
**

**
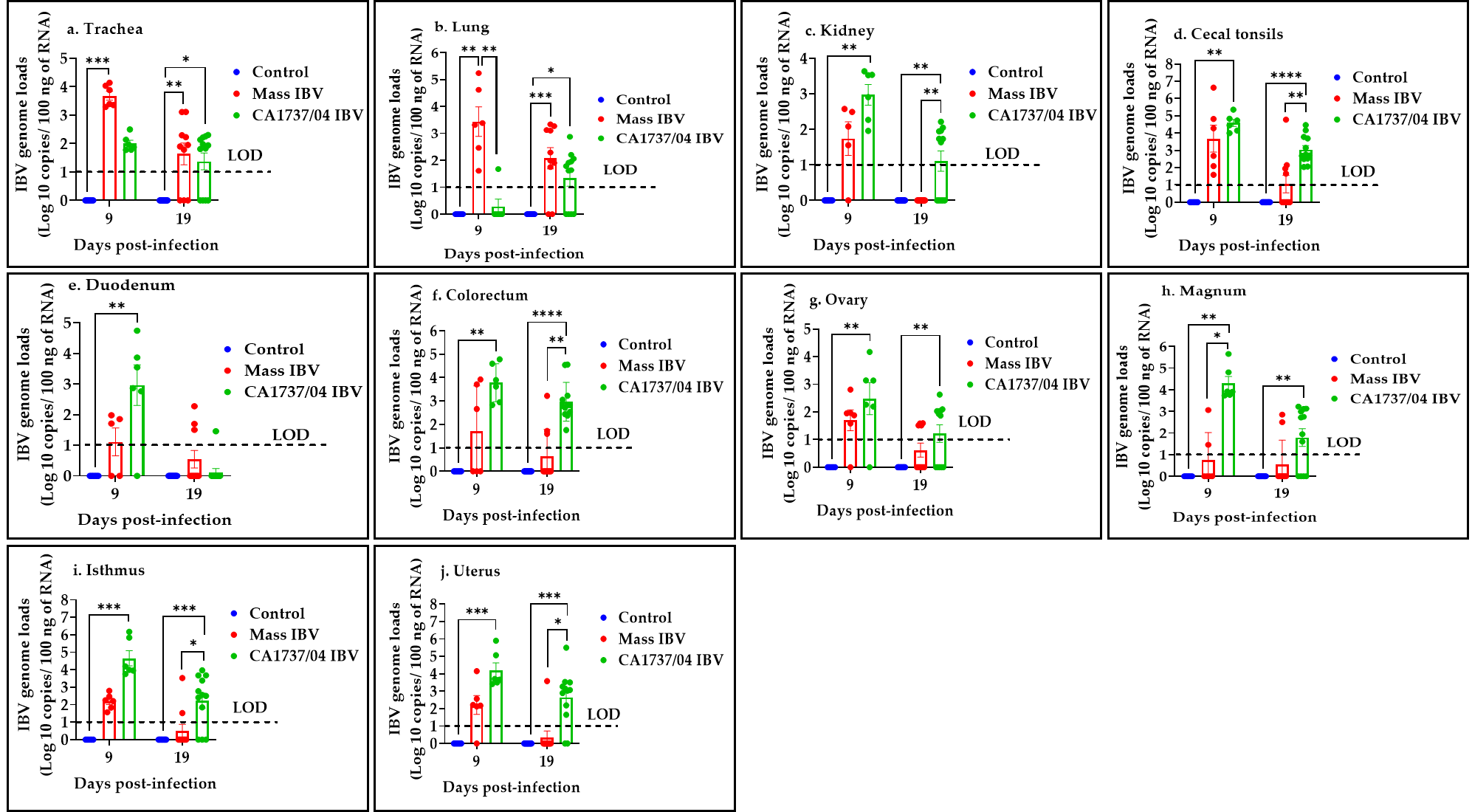
Fig. 6**


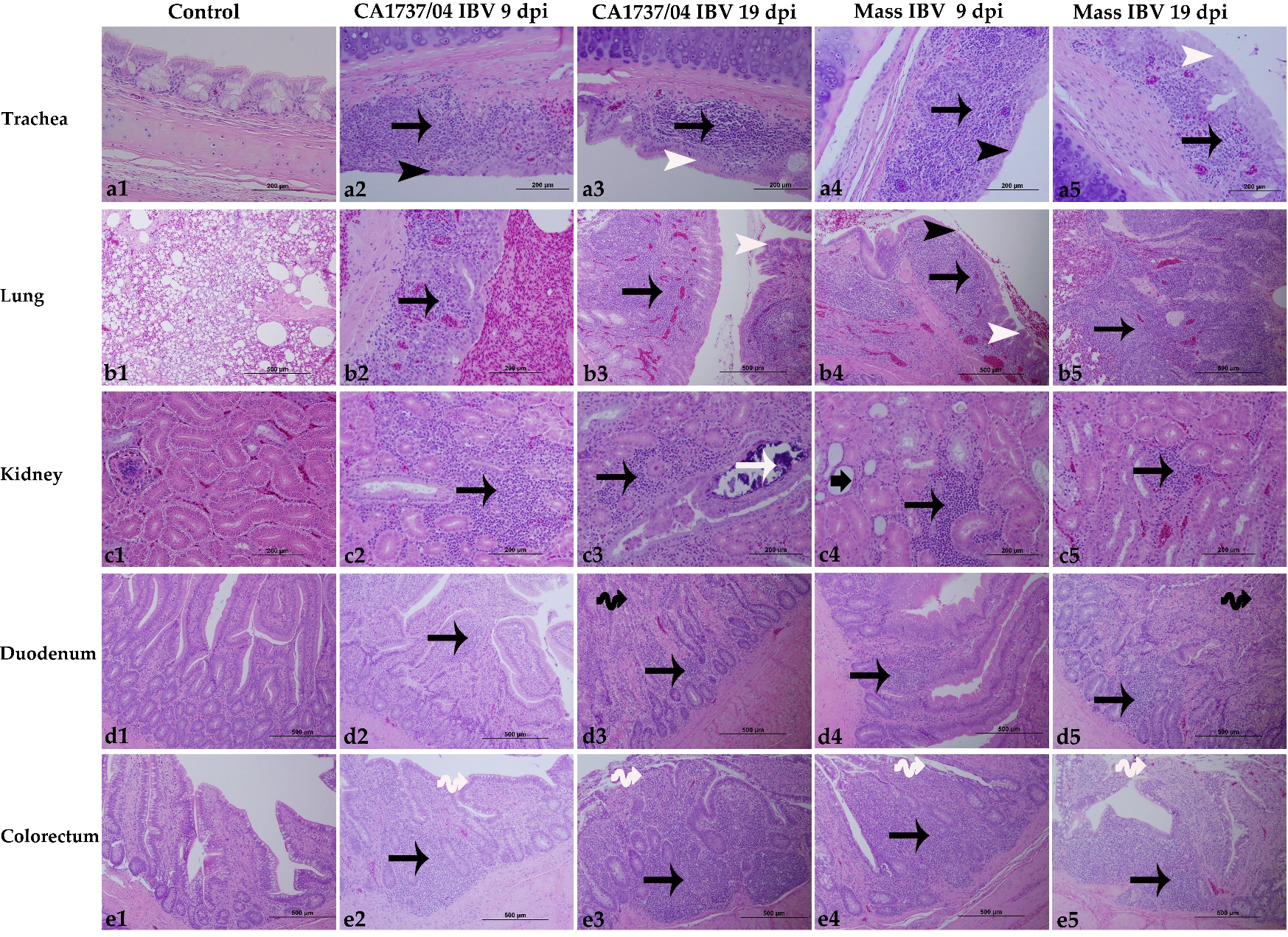
**Fig. 7**


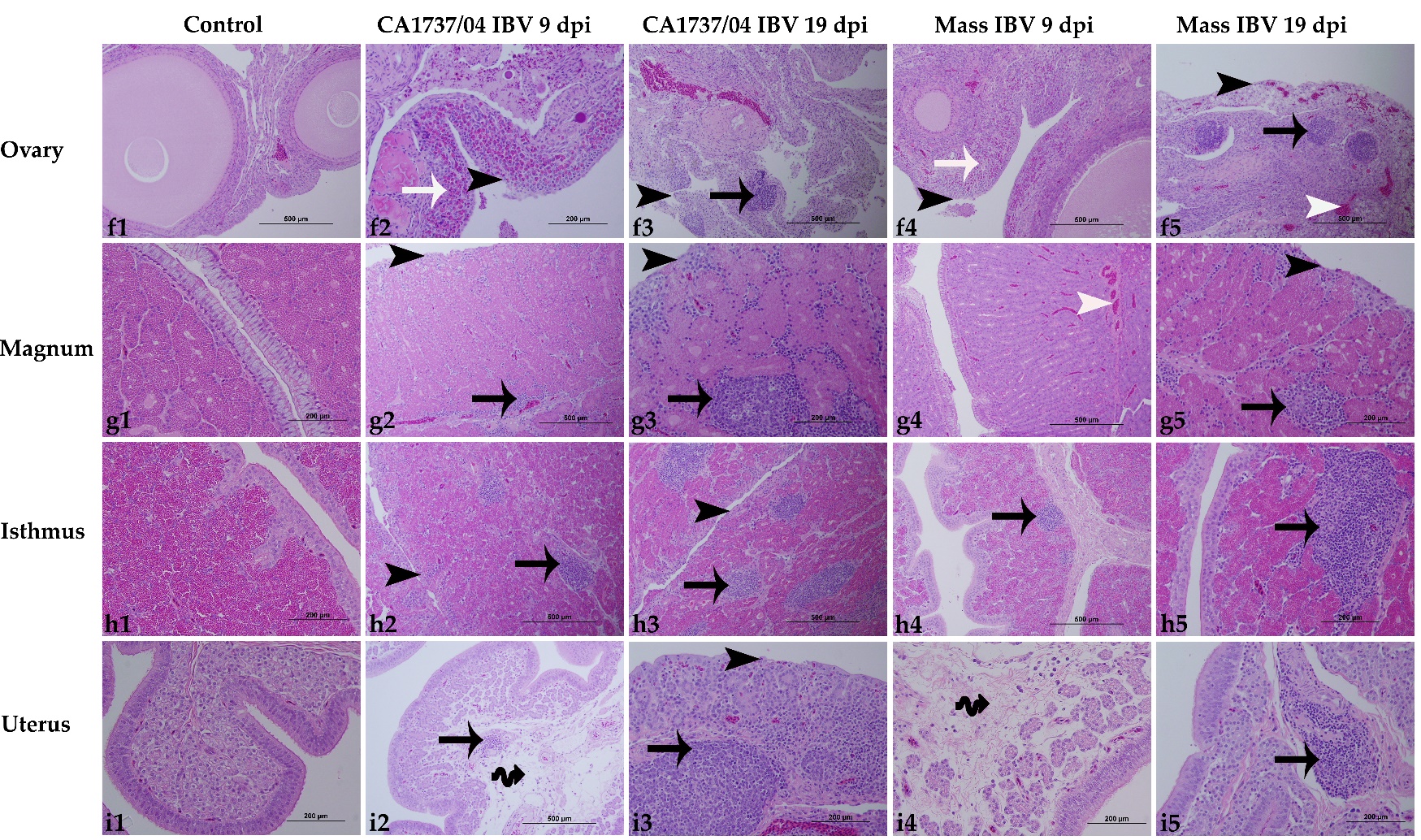
**Fig. 8**

**
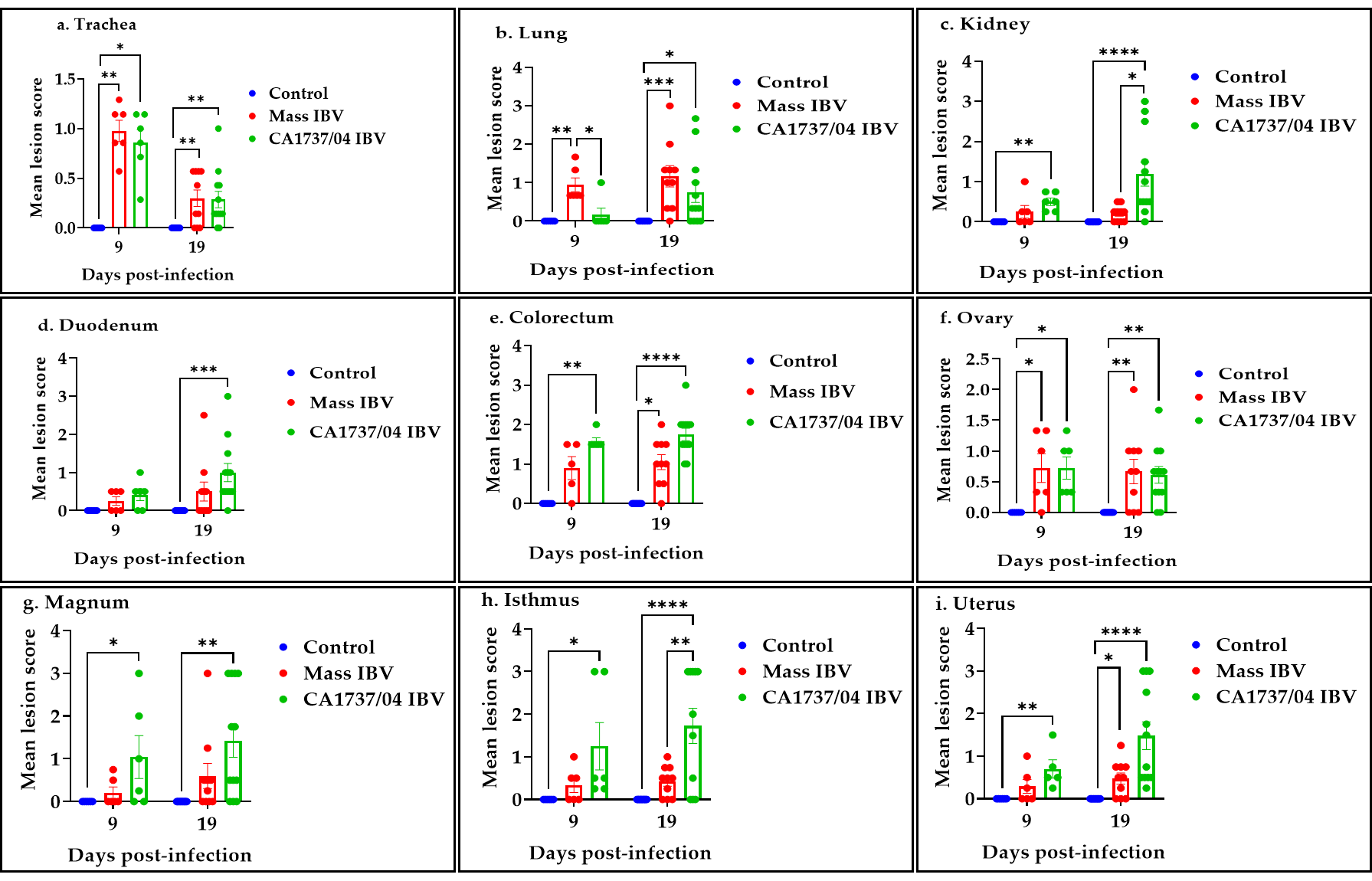
Fig. 9**


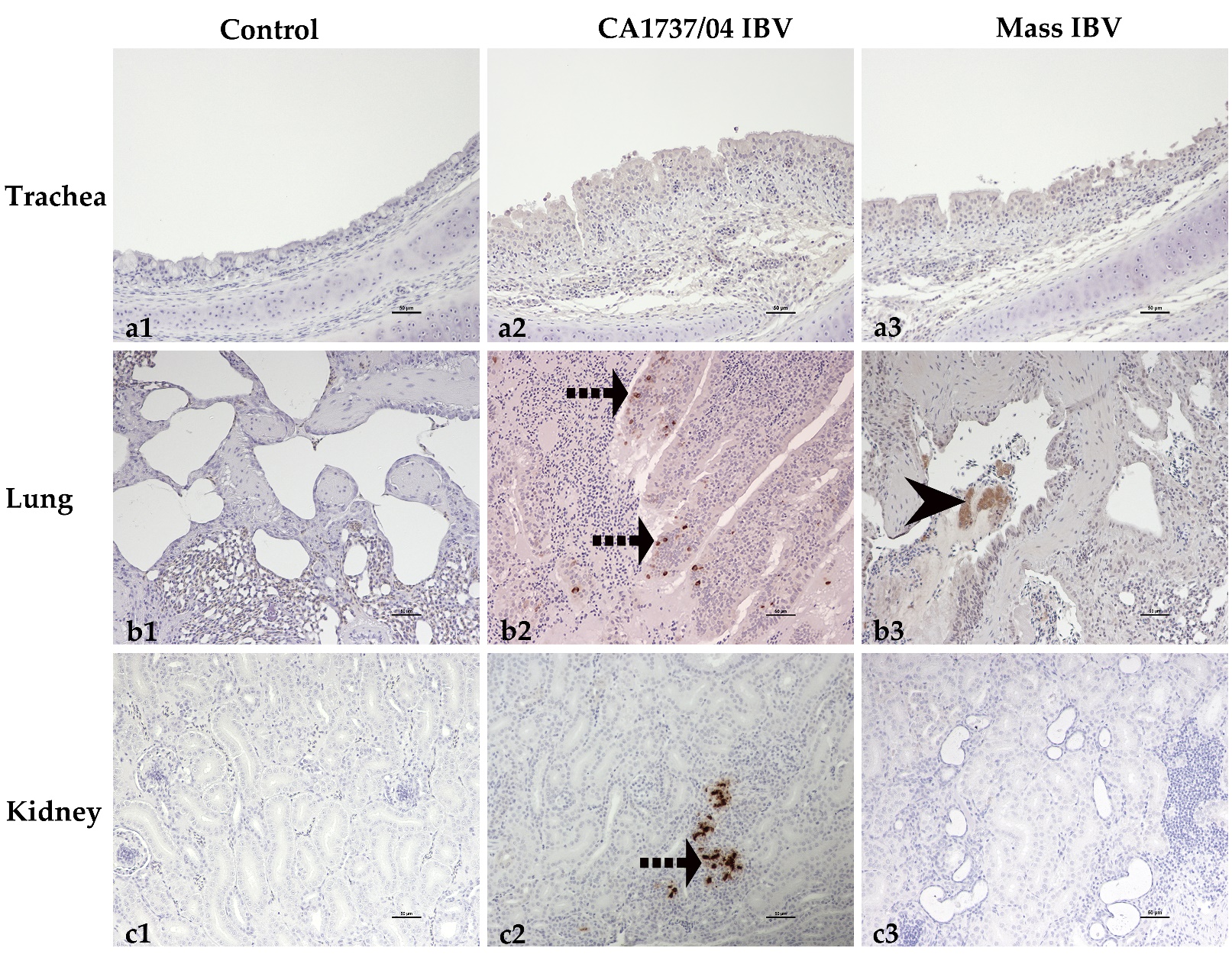
**Fig. 10**


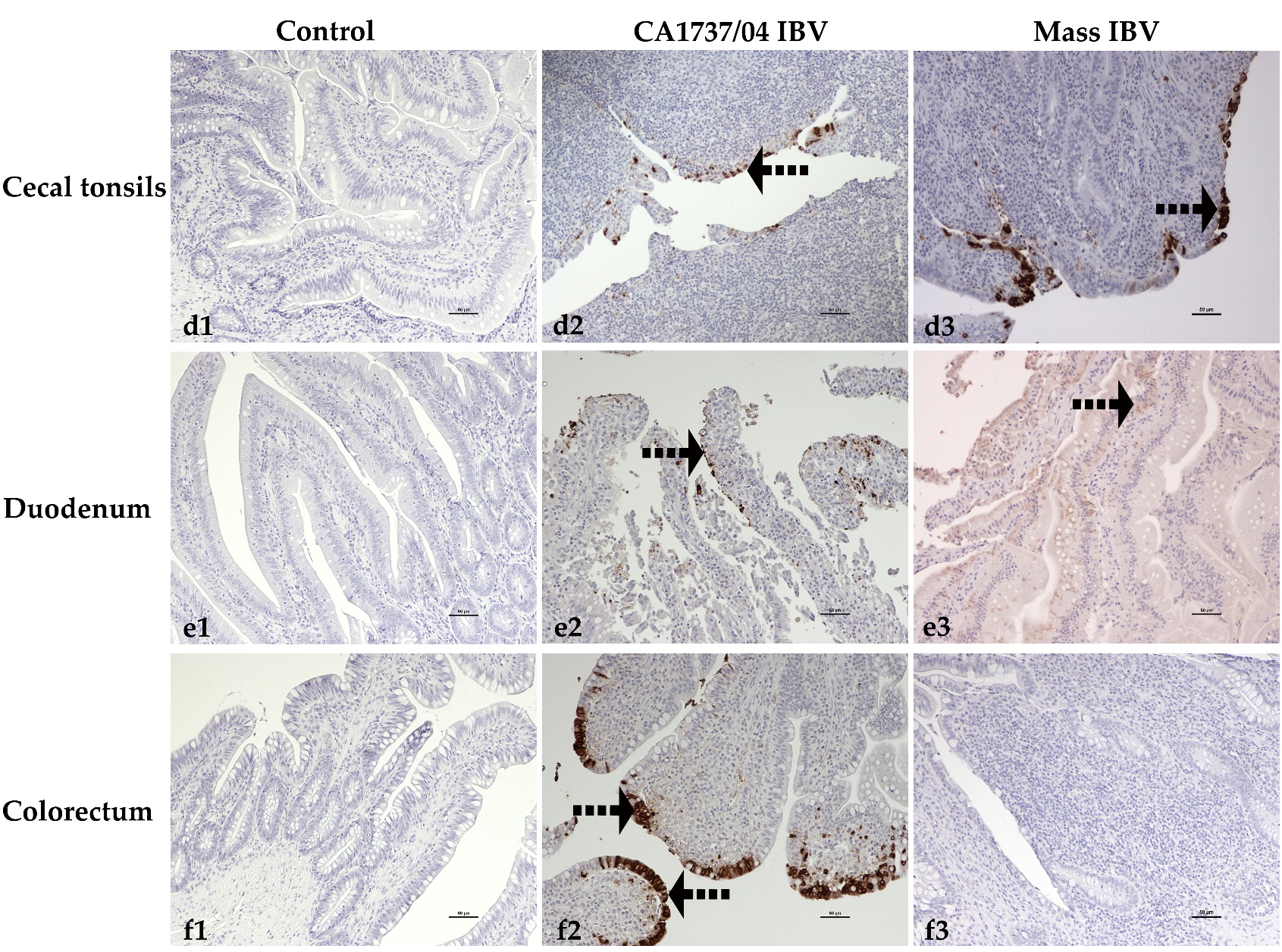
**Fig. 11**


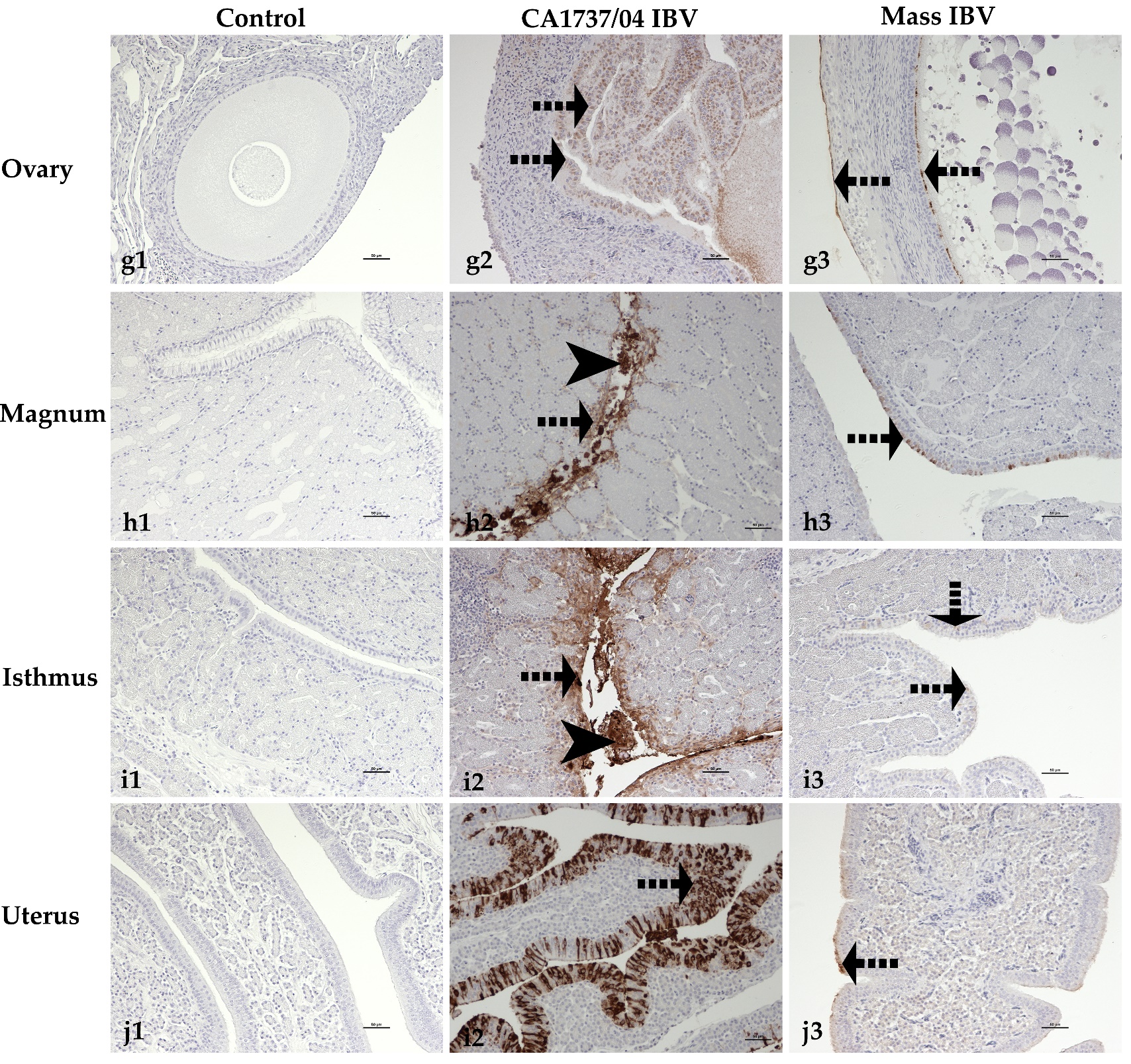
**Fig. 12**

**
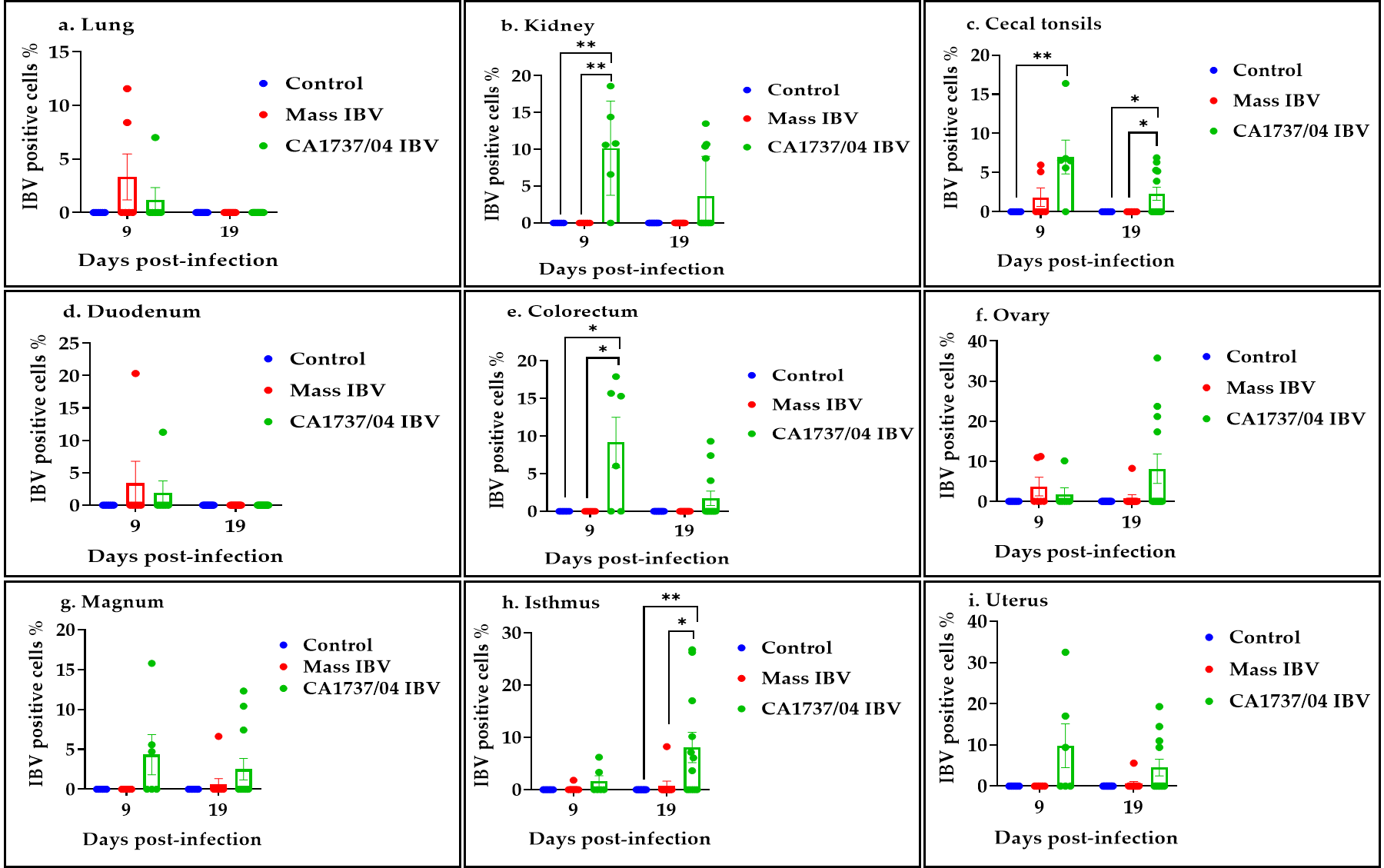
Fig. 13**

Supplement: Supplementary file 1 [file Data_Sheet_1.docx]
